# Aggregation of Aβ_40/42_ chains in the presence of cyclic neuropeptides investigated by molecular dynamics simulations

**DOI:** 10.1371/journal.pcbi.1008771

**Published:** 2021-03-12

**Authors:** Min Wu, Lyudmyla Dorosh, Gerold Schmitt-Ulms, Holger Wille, Maria Stepanova

**Affiliations:** 1 Department of Electrical and Computer Engineering, University of Alberta, Edmonton, Canada; 2 Laboratory Medicine and Pathobiology, University of Toronto, Toronto, Canada; 3 Department of Biochemistry, University of Alberta, Edmonton, Canada; 4 Centre for Prions and Protein Folding Diseases, Edmonton, Canada; 5 Neuroscience and Mental Health Institute, University of Alberta, Edmonton, Canada; Virginia Tech, UNITED STATES

## Abstract

Alzheimer’s disease is associated with the formation of toxic aggregates of amyloid beta (Aβ) peptides. Despite tremendous efforts, our understanding of the molecular mechanisms of aggregation, as well as cofactors that might influence it, remains incomplete. The small cyclic neuropeptide somatostatin-14 (SST_14_) was recently found to be the most selectively enriched protein in human frontal lobe extracts that binds Aβ_42_ aggregates. Furthermore, SST_14_’s presence was also found to promote the formation of toxic Aβ_42_ oligomers *in vitro*. In order to elucidate how SST_14_ influences the onset of Aβ oligomerization, we performed all-atom molecular dynamics simulations of model mixtures of Aβ_42_ or Aβ_40_ peptides with SST_14_ molecules and analyzed the structure and dynamics of early-stage aggregates. For comparison we also analyzed the aggregation of Aβ_42_ in the presence of arginine vasopressin (AVP), a different cyclic neuropeptide. We observed the formation of self-assembled aggregates containing the Aβ chains and small cyclic peptides in all mixtures of Aβ_42_–SST_14_, Aβ_42_–AVP, and Aβ_40_–SST_14_. The Aβ_42_–SST_14_ mixtures were found to develop compact, dynamically stable, but small aggregates with the highest exposure of hydrophobic residues to the solvent. Differences in the morphology and dynamics of aggregates that comprise SST_14_ or AVP appear to reflect distinct (1) regions of the Aβ chains they interact with; (2) propensities to engage in hydrogen bonds with Aβ peptides; and (3) solvent exposures of hydrophilic and hydrophobic groups. The presence of SST_14_ was found to impede aggregation in the Aβ_42_–SST_14_ system despite a high hydrophobicity, producing a stronger “sticky surface” effect in the aggregates at the onset of Aβ_42_–SST_14_ oligomerization.

## Author summary

Improper folding of proteins causes disorders known as protein misfolding diseases. Under normal conditions most proteins adopt particular folds, which allow them functioning properly. However, for reasons that are not yet fully understood, proteins may misfold and aggregate, forming deposits known as amyloid fibrils, which accumulate in the brain or other tissues. This process affects functioning of the nervous system, gradually causing loss of cognitive abilities. Alzheimer’s disease is one of the most common diseases from this group. A better understanding of the aggregation of peptides implicated in Alzheimer’s disease, known as amyloid beta (Aβ) peptides, may facilitate the development of treatments that ameliorate or prevent the disease. We use detailed molecular dynamics simulations to investigate the influence of somatostatin-14 (SST_14_), a cyclic neuropeptide that might be involved in the amyloidogenic aggregation of Aβ, on molecular processes occurring at the onset of Aβ aggregation. Results of these simulations explain how the presence of SST_14_ might alter pathways of aggregation of Aβ, shedding light upon the possible role of extrinsic factors in the aggregation at a molecular level.

## Introduction

Alzheimer’s disease (AD) is one of the most devastating neurodegenerative disorders of our time due to its high prevalence in the aging population, the challenges of early diagnosis and the lack of efficient therapeutics. AD is associated with the misfolding and formation of toxic aggregates of the amyloid β (Aβ) peptide [1,2]. It is believed that misfolding in spontaneously formed aggregates of Aβ chains eventually leads to a cascade of self-replicating misfolding events producing β-sheet rich amyloid deposits in the brain. However, mounting evidence suggests that relatively small prefibrillary oligomers rather than mature amyloid fibrils may be the primary toxic assemblies underlying the pathogenesis of the disease [2,3]. Insights into the molecular mechanisms of misfolding and aggregation, as well as co-factors that might influence these processes, remain incomplete.

Contributing to this status quo is a high level of heterogeneity in regards to both the building blocks and the architecture of early oligomeric assemblies. First, the Aβ peptide itself exists in multiple alloforms. Aβ_42_ comprises two C-terminal residues, I41 and A42, not present in Aβ_40_. Although the Aβ_40_ variant is more abundant than Aβ_42_, the latter is believed to be more pathogenic, primarily on the grounds of a relative increase in Aβ_42_ in the brain of AD patients, and higher rates of fibrillization *in-vitro* [4–6]. Second, Aβ oligomers are an ensemble of highly dynamic assemblies [7,8]. Experiments indicate that the initial small aggregates tend to adopt a largely unstructured morphology [9–11]. Quaternary structures without pronounced alignments of peptide chains were also predicted by molecular dynamics (MD) simulations for dimers of Aβ_40_ and Aβ_42_ [12–14] and larger multimeric Aβ aggregates [15,16]. Characterizations by a variety of biophysical methods *in vitro* suggest that initially unstructured oligomers may either undergo a transition into more ordered β-sheet-rich conformations, or mediate formation of β-sheet-rich assemblies [9–12]. However, the majority of early non-fibrillary aggregates dissociate into monomers rather than convert into fibrils [7,8], albeit they are potentially toxic [8,11]. Neither the detailed molecular mechanisms of aggregation, nor the accompanying misfolding or the specific structures and morphologies of the various non-fibrillary and pre-fibrillary assemblies are sufficiently understood. On theoretical grounds, it has been inferred that the aggregation process is driven by a competition of hydrophobic collapse and hydrogen bonding, with the former favoring unstructured conformations, and the latter giving rise to β-sheets [17–19]. The toxicity of non-fibrillary and pre-fibrillary oligomeric assemblies has been hypothetically linked to the exposure of hydrophobic groups and unpaired β-strands at their surfaces [9,20], often referred to as a “sticky surface” effect. Recent modeling studies [21,22] indicate that accumulation of subtle structural perturbations of early aggregates at the onset of the oligomerization process may result in distinct aggregation pathways at later, more advanced stages of Aβ oligomerization. Altogether, both experimental and theoretical evidence suggests that molecular events occurring early in the process of aggregation play a key role in determining both the structure and toxicity of Aβ oligomers [14].

The potential role of extrinsic factors in the aggregation and amyloidogenic conversion is a relatively under-explored aspect of immense importance for both the basic understanding of the aggregation process and the rational design of therapeutic strategies [23,24]. In particular, toxic Aβ*56 complexes have been hypothesized to require unknown cofactors for their assembly [25]. The cyclic neuropeptide somatostatin-14 (SST_14_) was reported to promote the proteolytic degradation of Aβ through neprilysin induction [26]. The level of SST_14_ in the brain decreases with ageing [26] and an accelerated decline is found in AD patients [27], potentially leading to an increase in steady-state Aβ levels. Recent experiments indicate that the cyclic neuropeptide somatostatin-14 (SST_14_) is the most selectively enriched peptide in human frontal lobe extracts that binds oligomeric Aβ_42_ aggregates [28]. Moreover, SST_14_’s presence was found to inhibit fibrillization of Aβ_42_ *in vitro*, while promoting formation of smaller oligomers, reminiscent of toxic Aβ*56 complexes [28,29]. Interestingly, Aβ_42_ but not Aβ_40_ peptides were prone to delayed fibrillization under the influence of SST_14_. This effect was also not observed upon replacement of SST_14_ with other cyclic neuropeptides, such as arginine vasopressin (AVP) [29]. Another recent biochemical study [30] investigated binding of SST_14_ to a specific membrane-associated Aβ_42_ tetramer, termed βPFO_Aβ(1–42)_. This report validated the ability of SST_14_ to selectively interact only with oligomeric assemblies of Aβ_42_. Consistent with previous data gathered with soluble Aβ_42_ oligomers [29], the authors reported that the binding interface between SST_14_ with βPFO_Aβ(1–42)_ may involve a central tryptophan (W8) within SST_14_. Whereas prior experiments [29] with soluble Aβ_42_ oligomers had pointed toward a contribution of the N-terminal half to the SST_14_ binding, the interactions with the membrane associated βPFO_Aβ(1–42)_ oligomer seems to primarily rely on residues 18–20 within the C-terminal half of Aβ_42_. Although at first glance these results may seem contradictory, the N-terminal half of Aβ_42_ is required for the formation of βPFO_Aβ(1–42)_, an observation that would have precluded the ability to detect binding of SST_14_ in assays that relied on truncated Aβ_18–42_ in prior binding studies. Both studies agreed that binding occurs predominantly to Aβ_42_ oligomers and is not observed with oligomers formed from Aβ_40_. Because analyses of [30] were restricted to a highly purified membrane-associated βPFO_Aβ(1–42)_, it remains unclear whether an alternative binding pose or binding stoichiometry would be available in soluble Aβ_42_ oligomers. Consistent with such a scenario, the binding constant for binding of SST_14_ to βPFO_Aβ(1–42)_ was approximately threefold higher than the previously observed Kd for binding of SST_14_ to soluble Aβ_42_ oligomers.

In order to elucidate how SST_14_ might influence the onset of Aβ oligomerization, we have performed all-atom molecular dynamics (MD) simulations of model systems containing mixtures of Aβ_42_ or Aβ_40_ monomeric peptides and SST_14_ molecules in explicit water. We also performed similar simulations for mixtures of Aβ_42_ peptides and AVP molecules as negative controls. We investigated early stages of aggregation in each system, and analyzed the structure and dynamics of the early self-assembled aggregates. For this purpose, we combined well-established structural analysis tools with a novel essential collective dynamics (ECD) method developed in our group [14,16], which allows to accurately identify persistent correlations of motion (dynamics) in a molecule or supramolecular system without exhaustive conformational sampling, based on an original fundamental concept [31,32]. The method allows analysing dynamics correlations between selected pairs of atoms; characterising main-chain flexibilities; and identifying domains of correlated motion within the same framework. The presence of SST_14_ was found to impede the aggregation in the Aβ_42_–SST_14_ mixtures despite having a high hydrophobicity. We attribute differences in the structures and dynamics of the Aβ_42_–SST_14_, Aβ_42_–AVP and Aβ_40_–SST_14_ systems to distinct tendencies of SST_14_ and AVP to (1) interact with specific regions of the Aβ chains; (2) develop hydrogen bonds with Aβ peptides; and (3) expose hydrophilic or hydrophobic groups to the solvent.

## Results

### Aggregation of Aβ peptides in the presence of small cyclic peptides

**Fig 1:**
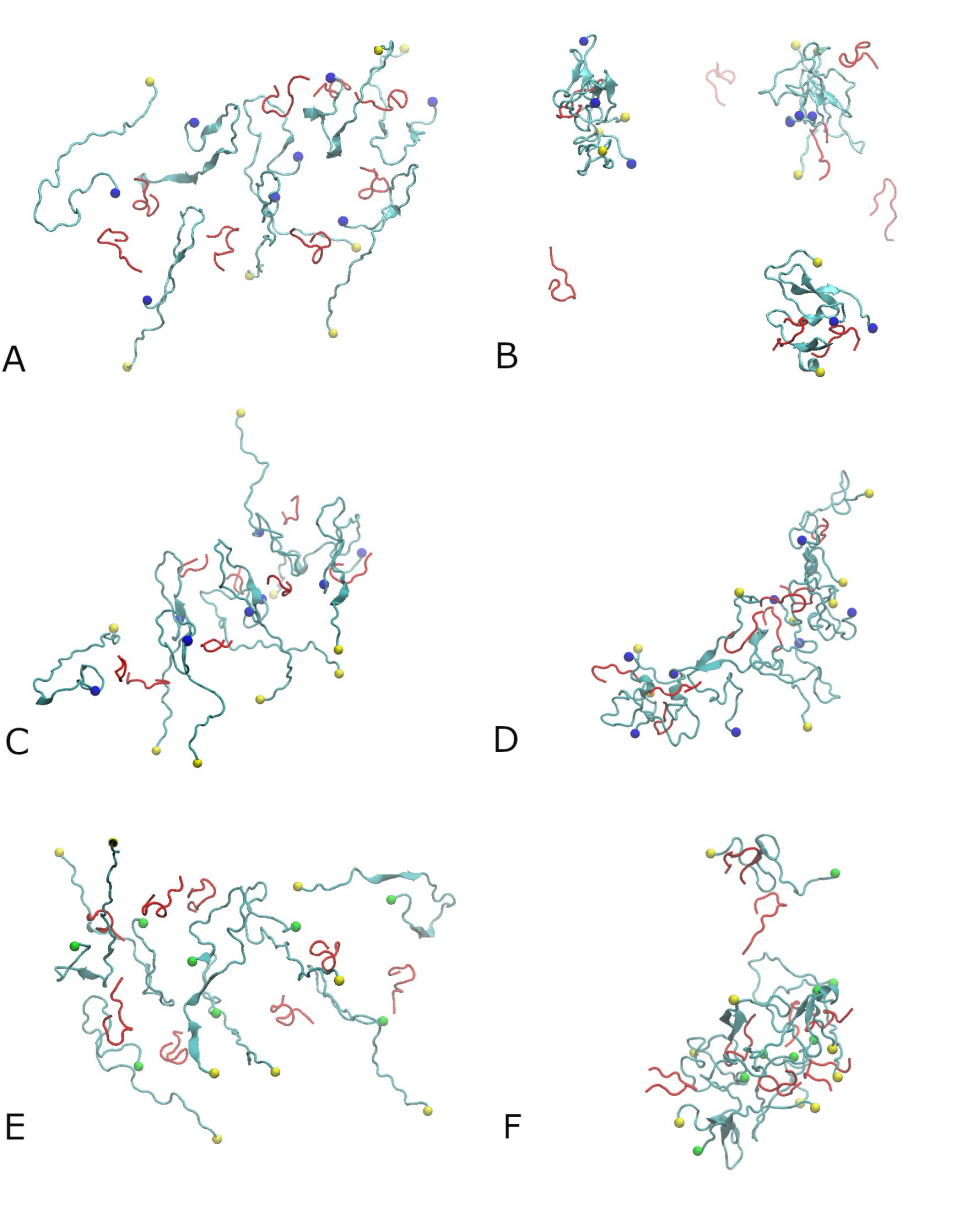
Eight Aβ_42_ monomers and eight SST_14_ molecules in water after equilibration (A), and the aggregates formed after 500 ns of MD simulation for system Aβ_42_-SST_14_-I (B). Eight Aβ_42_ monomers and eight AVP molecules in water after equilibration (C), and the octamer formed after 500 ns of MD simulation for system Aβ_42_-AVP-II (D). Eight Aβ_40_ monomers and SST_14_ molecules in water after equilibration (E), and the heptamer formed after 500 ns of MD simulation for system Aβ_40_-SST_14_-II (F). Aβ_42_ and Aβ_40_ chains are colored cyan, SST_14_ and AVP molecules are colored red. The C-termini of the Aβ_42_ chains are indicated by blue spheres and those of Aβ_40_ chains are shown as green spheres. The N-termini of Aβ_42_ and Aβ_40_ chains are indicated by yellow spheres.

Initially, eight Aβ_42_ or Aβ_40_ chains and eight SST_14_ or AVP molecules were placed in the simulation box in random positions, as outlined in the Methods section. In each system, the Aβ peptide chains are labelled A, B, C,…H, and the small cyclic peptides are labelled I, J, K,…P. Three independent 500 ns long MD simulations were conducted for each of the Aβ_42_-SST_14_, Aβ_42_-AVP and Aβ_40_-SST_14_ systems in explicit OPC water. To distinguish between the three trajectories, each of them were numbered by I, II, and III. Unless otherwise stated, the examples displayed below originate from trajectories Aβ_42_-SST_14_-I, Aβ_42_-AVP-II, and Aβ_40_-SST_14_-II. These trajectories were selected as representative examples of leading trends for each of the systems. Fig 1 shows the configurations for the three representative systems after the equilibration (see Methods), and after 500 ns production MD simulations. Additional details on the structures obtained in these trajectories can be found in S1 and S2 Figs in the Supporting Information. Table 1 summarizes the aggregation status in all nine systems after 500 ns MD simulations.

**Table 1:** Aggregation status in all trajectories after 500 ns MD simulations.

System	Aβ_42_-SST_14_	Aβ_42_-AVP	Aβ_40_-SST_14_
I	3Aβ_42_+2SST_14_; 3Aβ_42_+1SST_14_; 2Aβ_42_+2SST_14_; 3 free SST_14_.	6Aβ_42_+6AVP; 2Aβ_42_+2AVP.	7Aβ_40_+6SST_14_; 1Aβ_40_+1SST_14_; 1 free SST_14_.
II	4Aβ_42_+5SST_14_; 3Aβ_42_+2SST_14_; 1 free Aβ_42_; 1 free SST_14_.	8Aβ_42_+8AVP.	7Aβ_40_+6SST_14_; 1Aβ_40_+2SST_14_
III	5Aβ_42_+3SST_14_; 3Aβ_42_+3SST_14_; 2 free SST_14_.	6Aβ_42_+7AVP; 2Aβ_42_+1AVP.	7Aβ_40_+6SST_14_; 1 free Aβ_40_; 1 free SST_14_.

After equilibration of the Aβ_42_-SST_14_ system, Aβ chains were still relatively sparsely positioned, as shown in Fig 1A. After the 500 ns long production MD simulations, all three trajectories for this Aβ_42_-SST_14_ system developed self-assembled aggregates. Most aggregates contained two or three Aβ_42_ chains and one or two SST_14_ molecules, as illustrated in Figs 1B and S2A for system Aβ_42_-SST_14_-I. Somewhat larger aggregates were observed occasionally along with dimers and trimers. In system Aβ_42_-SST_14_-II four Aβ_42_ chains and five SST_14_ molecules formed an aggregate, and in system Aβ_42_-SST_14_-III five Aβ_42_ chains and three SST_14_ molecules formed an aggregate (Table 1). The SST molecules tend to attach to the surface of the Aβ_42_ oligomers such that their N- and C- terminal arms face the solvent.

When the eight SST_14_ molecules were replaced by AVP molecules, the starting configurations for the Aβ_42_-AVP system after equilibration also exhibited sparse positioning of the chains (Fig 1C). However, the final structures from the three trajectories comprise large aggregates composed of mixed Aβ_42_ and AVP (Table 1). The final configuration for system Aβ_42_-AVP-II is shown in Figs 1D and S2B. In this case, the largest aggregate is an octamer containing all eight Aβ_42_ chains and eight AVP molecules. In each of the other two Aβ_42_-AVP trajectories Aβ_42_ peptides formed a hexamer and a dimer. The hexamers contained 6 or 7 AVP molecules and at least one AVP was bound to each of the Aβ_42_ dimers.

For the Aβ_40_-SST_14_ system containing eight Aβ_40_ chains and eight SST_14_ molecules, all three trajectories developed aggregates composed of seven Aβ peptides and six SST_14_ chains. One Aβ_40_ chain remained free in each of the trajectories (Table 1). Fig 1E shows a representative configuration in system Aβ_40_-SST_14_-II after the equilibration, and Figs 1F and S2C depict this system after 500 ns of MD simulation. In this case, seven Aβ_40_ and six SST_14_ chains formed a curved semi-hollow structured oligomer. Six SST_14_ molecules are inserted inside the aggregate, whereas two SST_14_ are attached to the remaining free Aβ_40_ chain preventing its attachment to the larger aggregate.

Due to the putative importance of the C-terminal residues in defining the aggregation behaviour of Aβ peptides [4,19], the locations of the Aβ_42_ and Aβ_40_ C-termini are marked, respectively, by blue and green spheres in Fig 1. From Fig 1B and 1D it appears that the C-termini of Aβ_42_ peptides (blue spheres) tend to remain at the surface of the self-assembled aggregates in both the Aβ_42_-SST_14_–I and Aβ_42_-AVP-II systems. In the Aβ_40_-SST_14_–II system, the C-termini of several Aβ_40_ chains were buried inside the aggregate (Fig 1F). The N-termini of both Aβ_42_ and Aβ_40_ chains tend to remain at the surface of the oligomers in all systems.

**Fig 2:**
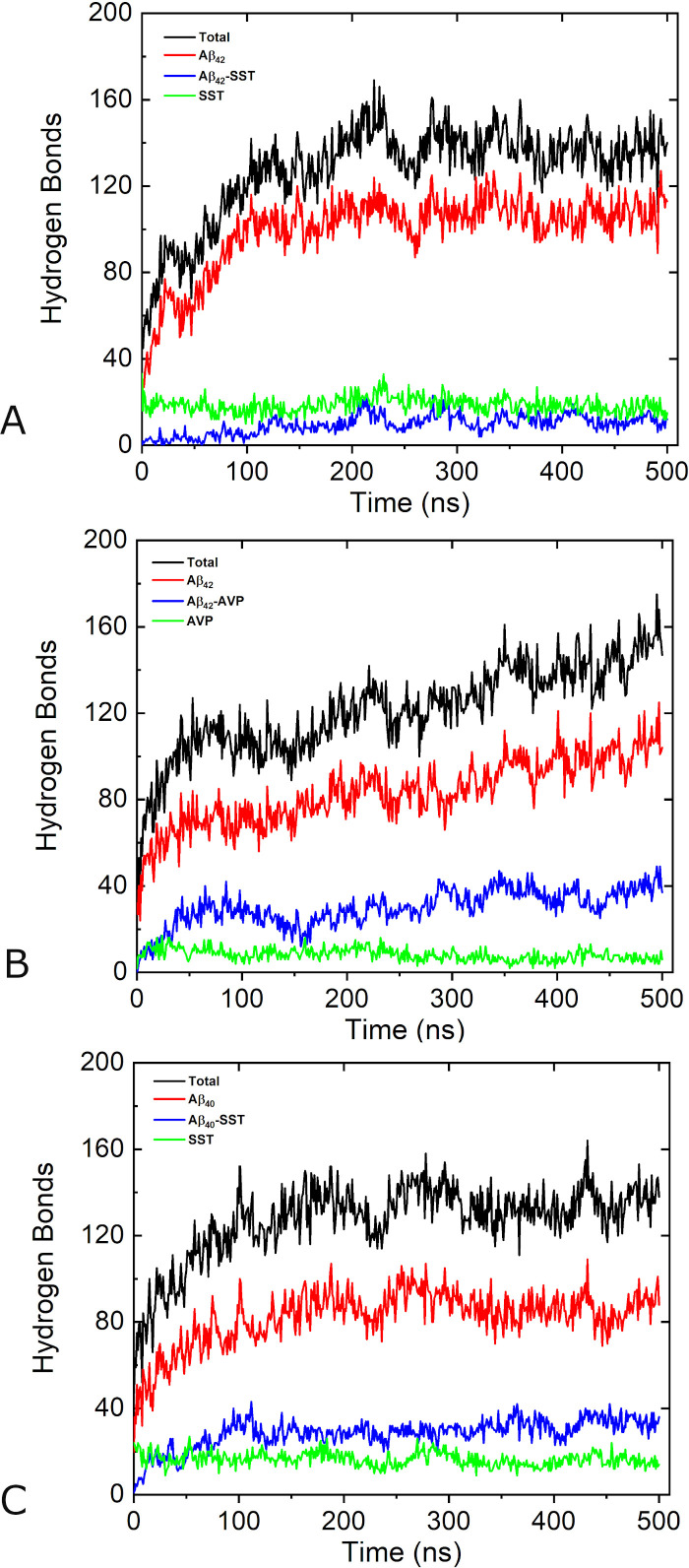
Number of hydrogen bonds for systems Aβ_42_-SST_14_-I (A), Aβ_42_-AVP-II (B), and Aβ_40_-SST_14_-II (C) during 500 ns production MD simulations. Hydrogen bonds between and across all chains are shown by black lines, hydrogen bonds between Aβ chains are shown by red lines, hydrogen bonds across Aβ chains and small cyclic peptides (SST_14_ or AVP) are shown by blue lines, and those between small cyclic peptides are shown by green lines.

The aggregation process is accompanied by a build-up of hydrogen bonds (HBs) in each system. Fig 2 shows the numbers of hydrogen bonds between and across all chains as functions of MD simulation time in systems Aβ_42_-SST_14_-I, Aβ_42_-AVP-II, and Aβ_40_-SST_14_-II. Additional details can be found in S1 Table, which lists average numbers of bonds in all 9 systems during the first and the last 20 ns of the 500 ns long simulations. In Fig 2 the black lines show how the total number of HBs between all chains evolved during the MD simulations. An increase in total HB number is observed in all systems. Overall, most HBs are formed over the first 200–250 ns of production MD simulations in all systems considered. An initial stage of fast build-up of hydrogen bonding ends at approximately 150–200 ns of the production MD simulation; then the build-up slows down although the number of HBs fluctuates considerably during subsequent simulations. In the system Aβ_42_-AVP-II a persistent increase in the total number of HBs continued throughout the entire simulation. By 500 ns this system developed more HBs than the other two systems, presumably due to the large size of the aggregate. As can be seen in S1 Table, the average number of HBs over three Aβ_42_-AVP trajectories at the end of the simulations was greater than similar averages in the other two systems as well.

In order to provide more detailed information on the hydrogen bonding, we analyzed the number of HBs between and across Aβ chains and the small cyclic peptides separately. In Fig 2, red lines represent the evolution of the number of HBs between Aβ chains in the three representative systems. As is evident from the figure, Aβ-Aβ bonds contribute a significant portion (60–80%) of the total number of HBs, and their time dependencies resemble those of the total number of HBs. According to S1 Table, at the beginning of the simulations the average numbers of Aβ-Aβ bonds were close in Aβ_42_-SST_14_ and Aβ_42_-AVP systems (49 and 52, respectively), and slightly above than in the Aβ_40_-SST_14_ system (44). In the course of the simulations, the average numbers of Aβ-Aβ bonds increased approximately twice in each of the systems.

The most dramatic differences were observed for hydrogen bonding across Aβ chains and small cyclic peptides (blue lines in Fig 2), and between small cyclic peptides (green lines in Fig 2). After equilibration and early into the production runs Aβ-Aβ, SST_14_-SST_14_, and AVP-AVP bonds were almost exclusively intra-molecular, due to distances between the chains in the initial structures. Examples illustrating this can be found in S1 Fig. During the first 20 ns after equilibrations, approximately 20 SST_14_-SST_14_ bonds in average were formed for each of two somatostatin-containing systems (S1 Table). In striking contrast, only ~9 AVP-AVP bonds have developed. In the course of the simulations the number of SST_14_-SST_14_ bonds changed only slightly adopting average values of 23 (for Aβ_42_-SST_14_ system) and 17 (for Aβ_40_-SST_14_ system) over the last 20 ns. At the same time, the average number of AVP-AVP bonds decreased barely remaining above ~7 over the last 20 ns. Such a large difference cannot be explained by different numbers of HB acceptor sites in SST_14_ and AVP (which are 24 and 16, respectively) and rather indicates differences in bonding behaviours of SST_14_ and AVP. As can be seen in S1 Table, during the first 20 ns of production simulations when the small cyclic peptides just started binding to Aβ chains, the average values of cross-species hydrogen bonds were in the proportion of Aβ_42_-AVP > Aβ_40_-SST_14_ > Aβ_42_-SST_14_. More specifically, the greatest average number of 12 cross-species bonds was found in Aβ_42_-AVP system, and the smallest average number of 5 cross-species bonds was observed in Aβ_42_-SST_14_ system. During the simulations the number of cross-species bonds increased by 3–5 times in each simulation, and exhibited a significant variability across individual trajectories (S1 Table and S3A Fig). However, the average number of Aβ_42_-AVP bonds remained approximately 2.4 times greater than the number of Aβ_42_-SST_14_ bonds. Similarly to the other two systems, the number of Aβ_40_-SST_14_ bonds varied across trajectories. In system Aβ_40_-SST_14_-II, for instance, the number of cross-species bonds is close to that in system Aβ_42_-AVP-II over significant parts of the two trajectories (S3B Fig). However, the average number of Aβ_40_-SST_14_ bonds is less than that of Aβ_42_-AVP (S1 Table and S3A Fig). Overall, regardless of the size and morphology of these aggregates, SST and Aβ tend forming less HBs than AVP and Aβ. This explains the large total number of hydrogen bonds in AVP-containing systems.

**Fig 3:**
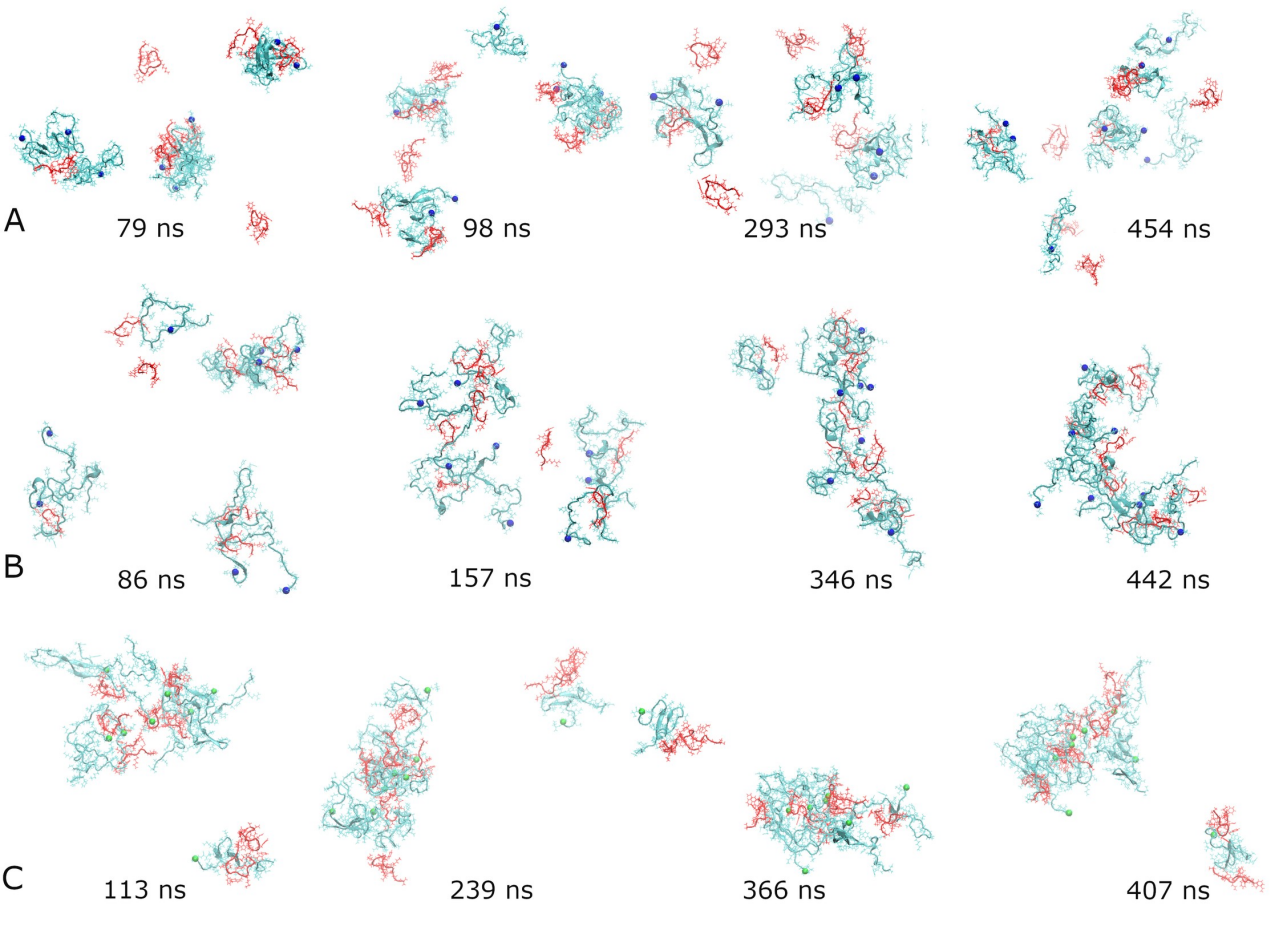
Snapshots of representative systems Aβ_42_-SST_14_-I (A), Aβ_42_-AVP-II (B), and Aβ_40_-SST_14_-II (C) corresponding to maxima or minima of the number of hydrogen bonds across Aβ chains and small cyclic peptides. In (A) maxima occur at 79 ns and 293 ns, and minima occur at 98 ns and 454 ns; in (B) maxima occur at 86 ns and 346 ns, and minima occur at 157 ns and 442 ns; and in (C) maxima occur at 113 ns and 366 ns, and minima occur at 239 ns and 407 ns, as illustrated in S3B Fig. The color code for peptides and small cyclic peptides is as in Fig 1. C-termini of Aβ_42/40_ chains are indicated by blue/green spheres.

In order to better understand how fluctuations in the number of hydrogen bonds relate to the morphologies, we examined the structures adopted by the three representative systems at several local maxima and minima of the cross-species HBs’ dependencies on time. These time dependencies are shown by blue lines in Fig 2. In addition, S3B Fig depicts the three time dependencies in one plot with solid dots indicating where the snapshots were taken. The corresponding structures are presented in Fig 3. The four snapshots for system Aβ_42_-SST_14_-I in Fig 3A illustrate an overall trend towards a greater compactness of several small aggregates. This is accompanied by transient detachment-adsorption of individual Aβ_42_ and SST_14_ chains, which appears to contribute to the observed fluctuations of the hydrogen bonding. For example, the structure at local minimum of 98 ns exhibits a monomeric (“free”) Aβ_42_ chain, three free SST_14_ molecules, and one SST_14_ molecule semi-detached from a small aggregate. Clearly there are more free chains than for 293 ns, where a maximum of HBs is observed. However, the local maximum of hydrogen bonding at 79 ns seems to exhibit a sparser morphology than the local minimum at 454 ns. We attribute this to the dynamic nature of the small aggregates and their propensity to restructure, which emerged as another factor contributing to the fluctuations. In the Aβ_42_-AVP system (Fig 3B) small aggregates self-assembled early in the simulations. The local maximum of hydrogen bonding at 86 ns already exhibits four aggregates and only one free AVP molecule. This is followed by a local minimum at 157 ns, where we observe a large but sparse aggregate, two smaller ones, and one free AVP. A significant decrease in the number of HBs can be attributed to the ongoing change in the aggregation status. Two aggregates observed at 346 ns are significantly more compact, and they exhibit a pronounced increase in hydrogen bonding. By 442 ns the two aggregates have merged into one C-shaped oligomer; however, the accompanying restructuring resulted in a loss in hydrogen bonding. The system Aβ_40_-SST_14_-II (Fig 3C) quickly developed two aggregates without any free chains resulting in a maximum in HB at 113 ns. Although the gross aggregation status was retained throughout the course of the simulation, the subsequent ups and downs of the hydrogen bonding suggest continuing restructuring of the aggregates.

After the 500 ns long MD simulations, the secondary structure of the self-assembled aggregates contains predominantly random coils and turns, as depicted in Fig 1. However, stable β-sheets are also observed. S4 Fig shows the secondary structure evolution over the 500 ns of three representative trajectories, and Table 2 lists percentages of stable β-sheet content in all 9 trajectories. The corresponding time dependencies of β-sheet content are given in S5 Fig. The greatest β-sheet content is found in two SST_14_–containing systems with a proportion of Aβ_40_-SST_14_ > Aβ_42_-SST_14_ > Aβ_42_-AVP in average over each set of three simulations (Table 2). In most cases we observe anti-parallel, intramolecular β-sheets in a hairpin conformation of the chain, often involving Aβ C-terminal residues 37–40 (in Aβ_42_ systems) or 36–39 (in Aβ_40_ system) as part of the β-sheets. Regardless of the system, most inter-chain β-sheets involve Aβ residues from the central region between positions 23 and 35, although specific locations of β-strands may vary. In several simulations (Aβ_42_-SST_14_-II and III, and Aβ_40_-SST_14_-II and III) β-strands from this region formed β-sheets with N-terminal residues of a different Aβ chain. The smaller SST_14_ and AVP molecules appeared less prone to contribute to the stable β-sheet content (see S4 Fig), although we have observed β-sheets involving them in eight out of nine trajectories. Populations of β-bridges and α-helices in all 9 trajectories are illustrated in S5 Fig. The corresponding averages are listed in S2 and S3 Tables. During the last 20 ns of the simulations, β-bridges are observed with average populations of approximately 4%, and α-/3-π-helices are observed with average populations of 1.5–3%.

**Table 2:** Stable β-sheet percentages in all trajectories averaged over the last 20 ns of the 500 ns MD simulations, their averages over three trajectories for each system, and standard deviations of the data.

System	Aβ_42_-SST_14_	Aβ_42_-AVP	Aβ_40_-SST_14_
I	11.54	8.16	2.25
II	4.53	2.12	12.00
III	4.39	4.70	8.83
Average	6.82	5.00	7.69
Standard Deviation	4.09	3.03	4.97

### ECD correlations of motion in self-assembled aggregates

**Fig 4:**
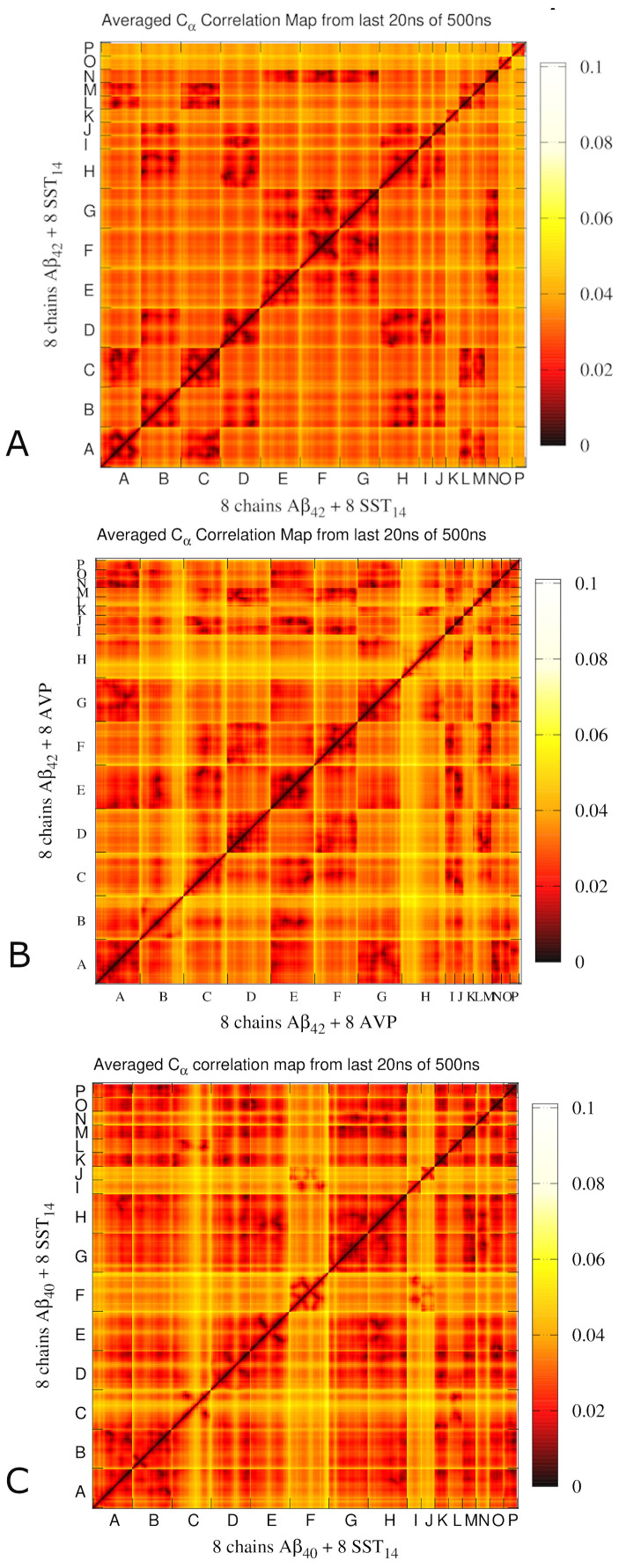
ECD C_α_ pair correlation maps for systems Aβ_42_-SST_14_-I (A), Aβ_42_-AVP-II (B) and Aβ_40_-SST_14_-II (C) averaged over the last 20 ns of 500 ns long MD trajectories. Stronger correlations of motion between C_α_ atoms are shown gradually by black, red, and orange colors, and weaker correlations are shown with white and yellow colors.

To probe collective motions of the Aβ_42_-SST_14_, Aβ_42_-AVP and Aβ_40_-SST_14_ systems, essential collective dynamics analyses (ECD) [16,31–35] have been performed. In the ECD method, principal eigenvectors of the covariance matrix calculated from MD trajectories are employed to characterize correlations of motion (dynamics) between atoms, as outlined in the Methods. Importantly, the method allows to reliably identify stable dynamical correlations without requiring exhaustive conformational sampling or convergence to equilibrium [31,32]. Fig 4 depicts ECD pair correlation maps [16,33,35] calculated for C_α_ atoms of all the chains from multiple 0.2 ns long segments of the MD trajectories and averaged over the last 20 ns of the simulations. To clarify the relation between pair correlations and morphologies, S6 Fig illustrates the three systems with all Aβ annotated similarly as in Fig 4.

For the system Aβ_42_-SST_14_-I, Aβ chains that exhibit strong inter-chain correlations in Fig 4A are involved in three oligomers: a dimer composed of Aβ chains A and C, which also contains SST_14_ molecules L and M; a trimer composed of Aβ chains B, D, and H, and SST_14_ molecules I and J; and a trimer composed of Aβ chains E, F, and G, and SST_14_ molecule N. Two pairs of Aβ chains, B-H and F-G, developed anti-parallel inter-chain β-sheets, whereas chains A, C, and F each developed intra-chain anti-parallel β-sheets. In addition, extensive regions of Aβ chains E, F, and G have wrapped around SST_14_ chain N and developed strong inter-molecular correlations with it. However, this interaction was not accompanied by a buildup of β-sheets (S6A Fig). The pair correlation map of the Aβ_42_-AVP-II system is shown in Fig 4B. Consistent with the structure depicted in S6B Fig, most Aβ chains are involved in strong inter-chain correlations of motion. The exceptions are semi-detached chains B and H, large parts of which exhibit a relatively independent motion from other Aβ units. The strongest inter-chain correlations are observed across Aβ chains A-E-G and D-F, which form two sides of a dumbbell-shaped aggregate. In addition, chain E is strongly correlated with C. These two chains connect two sides of the aggregate, and form a parallel inter-chain β-sheet in the region of the connection (S6B Fig). Each AVP molecule shows pronounced correlations with several Aβ chains, inter-connecting Aβ units in the aggregate. Fig 4C shows a pair correlation map for the Aβ_40_-SST_14_-II system. In this case, strong correlations are observed across six Aβ chains A, B, D, E, G, and H which have formed a large aggregate. The seventh chain, C, is correlated with the other six only through its N-terminal part due to its location at the periphery of the aggregate (S6C Fig). The C-terminus of chain B developed an inter-chain anti-parallel β-sheet with chain A, whereas the N-terminus of chain B formed an intra-chain anti-parallel β-sheet with its central region. Six SST_14_ molecules K, L, M, N, O and P are strongly correlated with the entire aggregate. Remarkably, these Aβ and SST_14_ chains exhibit inter-molecular correlations regardless of their proximity within the tertiary structure. The other two SST_14_ molecules, I and J, are correlated with the single detached chain F.

**Fig 5:**
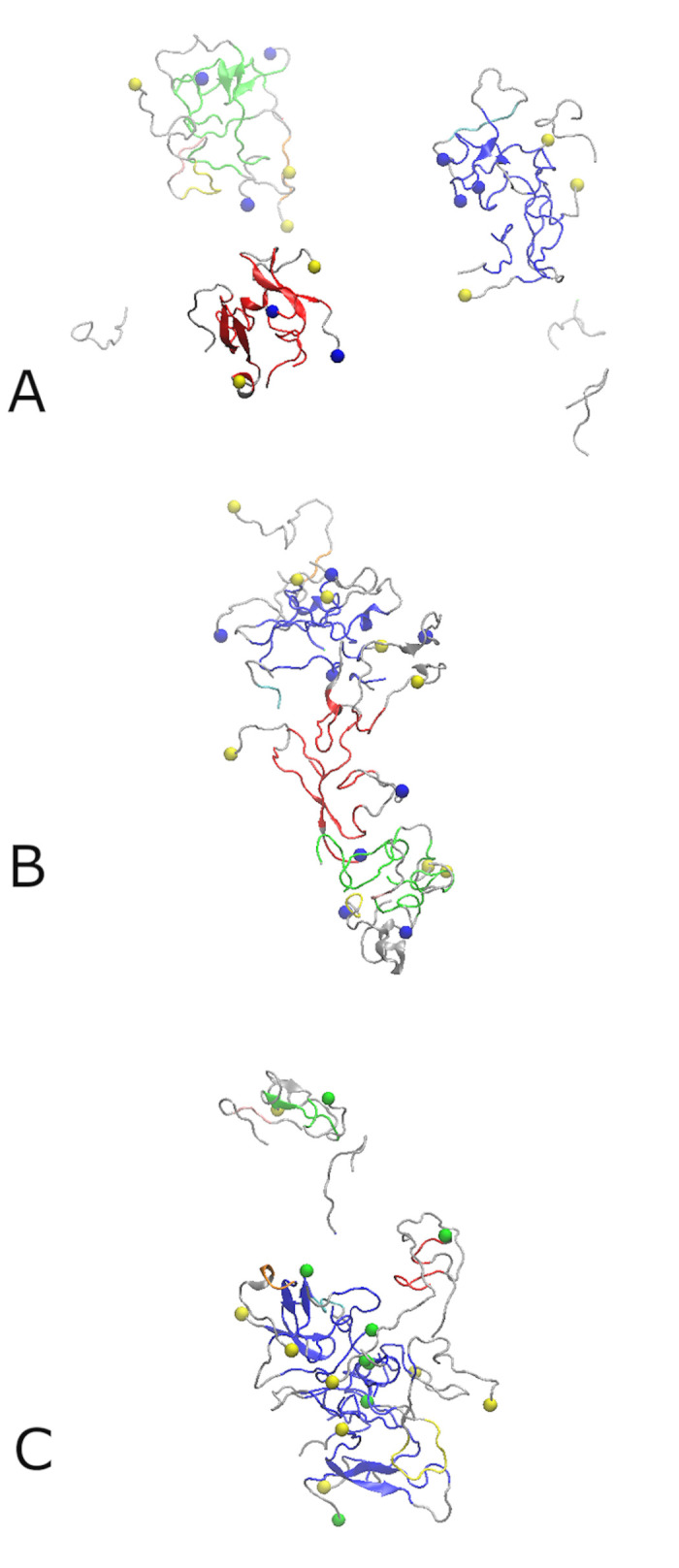
The largest ECD domains of correlated motion identified in the aggregates formed by the Aβ_42_-SST_14_–I system (A), the Aβ_42_–AVP-II system (B), and the Aβ_40_-SST_14_–II system (C). The dynamics domains are colored blue, red, green, yellow, cyan, orange, and pink in the order of decreasing size. Off-domain regions are colored gray. Blue and green spheres denote the C-termini of Aβ_42_ and Aβ_40_ chains, respectively. Yellow spheres denote the N-termini of the Aβ chains.

The dynamics correlations identified in the ECD framework can also be visualized in the form of domains of correlated motion [16,31,32], which represent relatively rigid parts of the system consisting of atoms moving coherently. Fig 5 shows the largest domains of correlated motion color-mapped onto the secondary structure of self-assembled aggregates in the three systems. The domains are colored blue, red, green, yellow, etc. in the order of decreasing size. In the Aβ_42_-SST_14_–I system shown in Fig 5A, the three largest domains of correlated motion colored blue, red, and green are located in central parts of the three aggregates. These domains include both Aβ and SST_14_ chains that are strongly inter-correlated in Fig 4A, and contain most of the β-structure. Peripheral regions, which often include termini, tend to exhibit relatively independent dynamics. In the case of the Aβ_42_–AVP-II system shown in Fig 5B we observe three large domains as well; however, all three are included in a single dumbbell-shaped aggregate. The domain colored blue contains Aβ chains A and G and AVP chains N and O, whereas the domain colored green consists mainly of Aβ chain F and AVP chain I. These two domains are located at the core of two sides of the “dumbbell”, which are held together through the mediation of the third domain (colored red), which includes parts of Aβ chains C and E along with AVP molecule J. In the Aβ_40_-SST_14_-II system depicted in Fig 5C, the largest domain of correlated motion (colored blue) is located in the aggregate and consists primarily of Aβ chains A, B, E, G, and H; SST_14_ chains M, N, O, and P; and parts of several other chains. This domain contains most β-sheets of the aggregate. The second largest domain (colored red) contains the C-terminus of Aβ chain C and a part of SST_14_ chain L.

### Main-chain flexibility profiles

**Fig 6:**
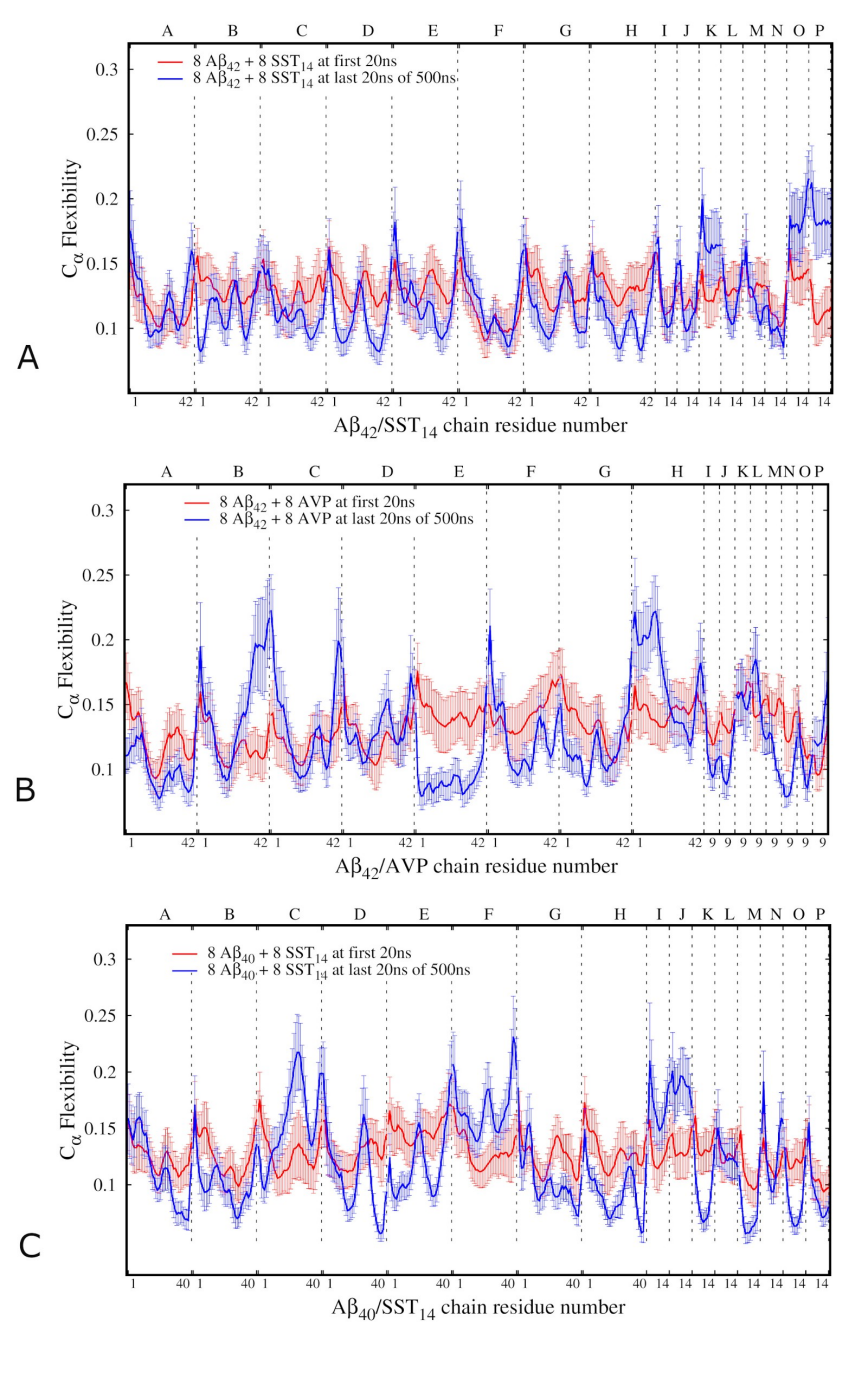
ECD main chain flexibility profiles of the Aβ_42_-SST_14_–I system (A), Aβ_42_–AVP-II system (B) and Aβ_40_-SST_14_-II system (C). Red and blue lines indicate, respectively, the average flexibilities for the first 20 ns and the last 20 ns of the production MD simulations. The labels on top of the horizontal axis denote the chains, and those at the bottom of the horizontal axis denote the residue numbers for each chain.

Within the same ECD framework, main-chain flexibility profiles [16,33–35] can be calculated for all the peptides in each system. High levels of the flexibility descriptor usually correspond to flexible loops and termini, whereas minima indicate more restrained regions. The main-chain flexibility profiles averaged over the first and the last 20 ns of the MD simulations for the Aβ_42_-SST_14_, Aβ_42_-AVP and Aβ_40_-SST_14_ systems are shown in Fig 6.

In the Aβ_42_-SST_14_ system, most Aβ chains show a decreased flexibility at the last 20 ns of production MD simulation compared to the first 20 ns (Fig 6A), as one could expect since oligomerization limits mobility of the chains. The flexibility profiles of individual Aβ chains tend to adopt oscillating shapes with several maxima and minima. Although by the end of the simulations N- and C-terminal ends of the chains remain flexible, deep minima developed within 10–12 residues from one or both of the termini in most chains. This is especially the case for chains B, D, and H, which have formed a trimeric self-assembled aggregate (S6A Fig). In central regions of the Aβ chains the flexibility profiles are often M-shaped with two maxima separated by a minimum (chains B, C, and E). However in chains A, D, and G the central regions exhibit flexible loops located at the periphery of the respective aggregates. Minima of main-chain flexibility often coincide with locations of stable β-strands. In particular, this is the case for the flexibility minima in chains A, B, C, F, G and H, where stable β-strands are found (see S6A Fig). However, not all flexibility minima are necessarily associated with the presence of β-structure. For example, we do not observe stable β-sheet content in chains D and E, which exhibit pronounced minima of main-chain flexibility. A trend of adopting lower main-chain flexibility in central regions in comparison to the N- and C-termini is observed in most SST molecules in the Aβ_42_-SST_14_ system. Moreover, at the end of the simulation the central regions of the SST molecules I, J, L, and M exhibit comparable flexibilities with parts of the Aβ chains B, D, H, A, and C, with which they directly interact. Interestingly, the SST molecules K, O, and P adopted a higher flexibility during the last 20 ns in comparison to the beginning of the simulation. An analysis of the trajectory indicated that initially these molecules were surrounded by neighbouring Aβ_42_ chains (S1A Fig), which somewhat limited their motion. By the end of the simulations the Aβ_42_ chains and other SST_14_ molecules formed three aggregates, whereas the molecules K, O, and P remained free. Eventually, remaining in solution resulted in a less constrained motion for these SST molecules by the end of the simulation.

Fig 6B depicts the main-chain flexibility profile for the Aβ_42_-AVP system. After the equilibration most chains exhibit relatively uniform flexibility patterns, whereas by the end of the simulation pronounced differences emerged across the various chains. Both termini of chains B and C, as well as the N-terminus of chain F, and the C-terminus of chain G adopted higher flexibility levels in the course of the simulation, which can be explained by their location at the periphery of the self-assembled aggregate. In contrast, extensive regions of chains A, E, F, and G, which are located at the core of the three domains of correlated motion (Figs 5 and S6B), exhibited low flexibility. Flexibilities of AVP molecules tended to follow those of the Aβ chains with which they interacted over the last 20 ns of the simulation.

The main-chain flexibility profile of the Aβ_40_-SST_14_ system is shown in Fig 6C. Extensive regions of Aβ chains A, B, D, G and H, which are located in the interior regions of the aggregate, developed a decrease in flexibility during MD simulation. In contrast, chain F and most of chain C adopted relatively high flexibilities due to their less constrained positions. Central regions of SST_14_ molecules K, M, O, and P exhibited relatively low main-chain flexibility over the last 20 ns of the MD simulation. These chains were actively involved in the oligomerization process, which resulted in their insertion inside the self-assembled aggregate where they became a part of the largest domain of correlated motion (see also Figs 5C and S6C). SST_14_ molecules I and M, which attached to the free Aβ chain F, exhibited high flexibility during last 20 ns of the simulations.

Table 3 lists the main-chain flexibility of the Aβ_42_ or Aβ_40_ C-termini averaged over eight Aβ chains in each of the three MD trajectories in each system. The table also lists the results of averaging over the three MD trajectories for each system. Further details of C-terminal flexibilities in individual Aβ chains can be found in S4 Table in the Supporting Information. As Table 3 illustrates, the average flexibility of the C-termini is greater in the AVP-containing systems than in the SST-containing systems. AVP-containing systems also exhibit the greatest variation of C-terminal flexibility across individual trajectories. Interestingly, the average flexibility of the C-termini in the Aβ_40_-SST_14_ system is very close to that in the Aβ_42_-SST_14_ system (Table 3), despite the absence of two hydrophobic residues at the C-terminus of Aβ_40_ and the fact that the Aβ_40_ C-termini are often buried inside the Aβ_40_-SST_14_ aggregates.

**Table 3:** ECD main-chain flexibilities of the C-termini averaged over eight Aβ_42_ or Aβ_40_ chains in each trajectory over the last 20 ns of MD simulations, their averages over three trajectories for each system, and standard deviations of the data.

System	Aβ_42_-SST_14_	Aβ_42_-AVP	Aβ_40_-SST_14_
I	0.150	0.152	0.153
II	0.155	0.170	0.147
III	0.146	0.164	0.141
Average	0.150	0.162	0.147
Standard Deviation	0.005	0.009	0.006

### Intermolecular interactions in self-assembled aggregates

**Fig 7:**
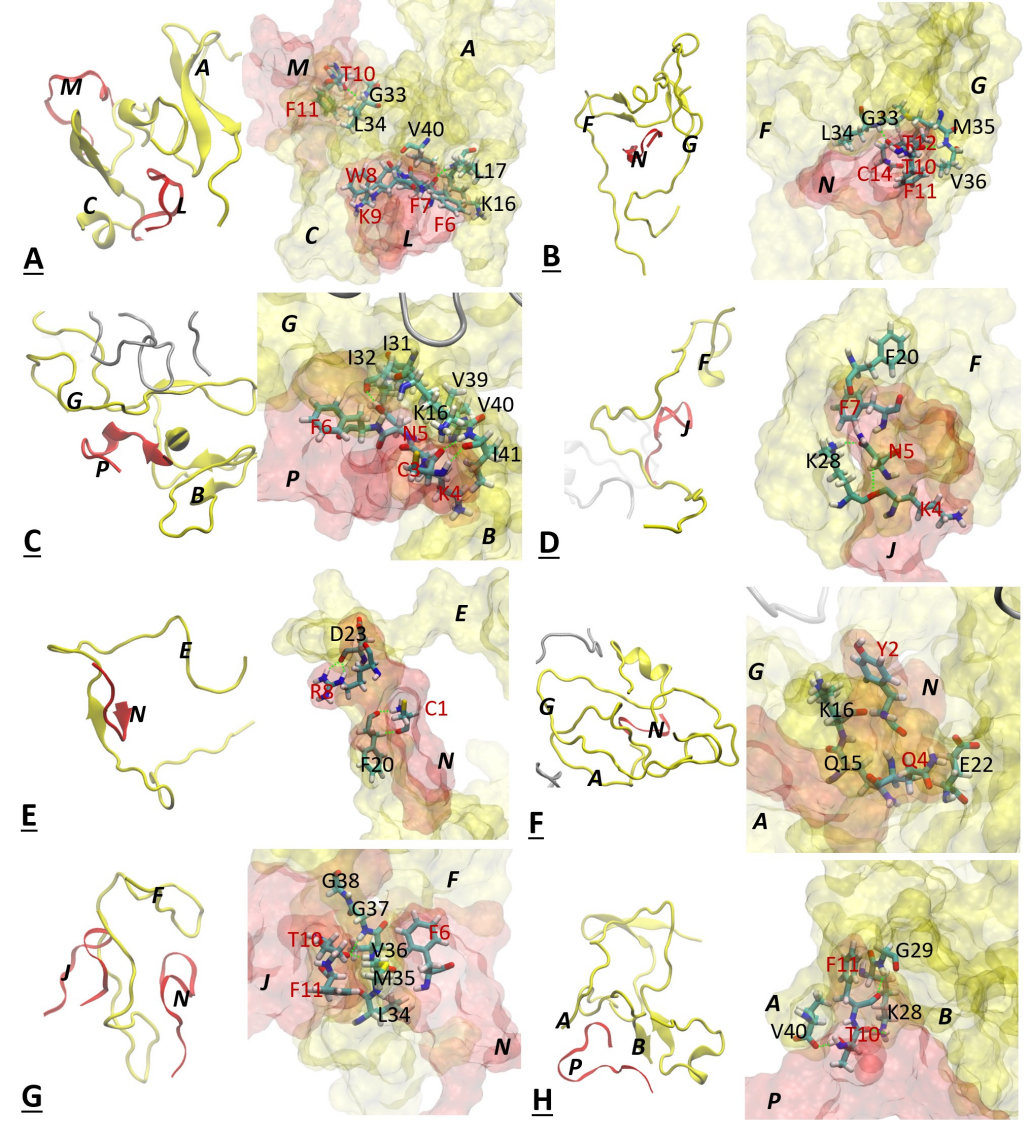
Close-ups of interactions between Aβ chains and small cyclic peptides in systems Aβ_42_-SST_14_-I (A,B), Aβ_42_–SST-II (C,D), Aβ_42_-AVP-I (E), Aβ_42_-AVP-II (F), Aβ_40_-SST_14_-I (G) and Aβ_40_-SST_14_-II (H). Aβ_42_ or Aβ_40_ chains are colored yellow, and SST_14_ or AVP molecules are colored red. Aβ residues are annotated with black letters and small cyclic peptide residues are annotated with red letters. Hydrogen bonds are depicted by green dashed lines. The close-ups illustrate interactions resulting in the strongest ECD pair correlations averaged over the last 20 ns of 500 ns long MD trajectories (Fig 4).

In order to elucidate the molecular mechanisms behind the influence of the small cyclic peptides onto the self-assembled aggregates, we identified the SST_14_ and AVP molecules that exhibited the strongest inter-molecular dynamics correlations by averaging over the last 20 ns, in each MD trajectory and for all the Aβ_42_-SST_14_, Aβ_42_-AVP and Aβ_40_-SST_14_ systems. Fig 7 depicts close-ups of the identified highest correlated regions involving small cyclic peptides and Aβ chains.

Fig 7A and 7B illustrate, respectively, the interactions between an Aβ dimer (A+C) and the SST chains L and M attached to it, and between Aβ chains F+G and the SST chain N in system Aβ_42_-SST_14_-I. Fig 7C shows the interactions between Aβ chains G and B and SST chain P, and Fig 7D illustrates the interactions between Aβ chain F and SST chain J in system Aβ_42_-SST_14_-II. In these examples, the strongest inter-molecular correlations involve pairs of residues: K16-F7, G33-T10, and V40-W8, (Fig 7A), G33-T12 and M35-T10 (Fig 7B), I32-N5 and V40-K4 (Fig 7C), and K28-N5 (Fig 7D). Remarkably, the SST residues involved in strong correlations with Aβ_42_ are often hydrophobic (F7, W8), or carry a long hydrocarbon side chain (K4), and/or have hydrophobic neighbors such as F6 or F11. Similarly, the pairing Aβ residues often carry hydrophobic groups (M35, V40) and/or have hydrophobic neighbors (L17, I31, I32, L34, I41). These hydrophobicity-driven interactions of side chains are accompanied by backbone-backbone hydrogen bonding, for example L16-F7, G33-T10 (Fig 7A), L34-T12 and V36-T10 (Fig 7B), or I41-K4 (Fig 7C). Complementary to hydrophobic interactions and hydrogen bonding, we also detected π-π interactions between residue F20 of Aβ_42_ and F7 of SST_14_ (Fig 7D).

Next, Fig 7E and 7F depict the strongest interactions between Aβ_42_ and AVP molecules identified in systems Aβ_42_-AVP-I and Aβ_42_-AVP-II. In the first system illustrated by Fig 7E, AVP residue R8 from chain N developed strong dynamics correlations with D23 of the Aβ_42_ chain E. Moreover, residue C1 developed contacts with residue F20 located nearby, although this interaction is somewhat weaker. In this case, both AVP residues have formed hydrogen bonds with their Aβ_42_ counterparts resulting in formation of a cross-species β-sheet (Fig 7E). Fig 7F illustrates the strongly correlated pair consisting of Aβ_42_ residue K16 (chain G) and AVP residue Y2 along with a weaker interaction of closely positioned pair of E22 (chain A) and Q4. Despite an involvement of hydrophobic contacts between Y2 and a hydrocarbon group of K16, electrostatic forces rather than hydrophobicity appear to play a role in this interaction.

Interactions between Aβ_40_ chains and SST_14_ molecules are illustrated with examples from system Aβ_40_-SST_14_-I (Fig 7G) and system Aβ_40_-SST_14_-II (Fig 7H). In system Aβ_40_-SST_14_-I, the strongest dynamics correlations involve residues M35, V36, and G37 from Aβ chain F and residues T10 and T11 from SST chain J. Backbones of residues M35 and G37 developed hydrogen bonds with F11 and T10, respectively, whereas residue V38 formed a hydrogen bond with T10 as well as hydrophobic interaction with F11. In system Aβ_40_-SST_14_-II, Aβ residues V40 (chain A) and K28 (chain B) exhibit strong dynamic correlations with residues T10 and F11 from SST chain P, respectively. All these interactions involve hydrogen bonding (Fig 7H). Strong correlations that involve hydrophobic contacts are less frequent in the Aβ_40_-SST_14_ system than in the Aβ_42_-SST_14_ system, evidently due to the lack of two C-terminal residues I41 and A42 in Aβ_40_.

### Solvent accessibility of the aggregates

**Fig 8:**
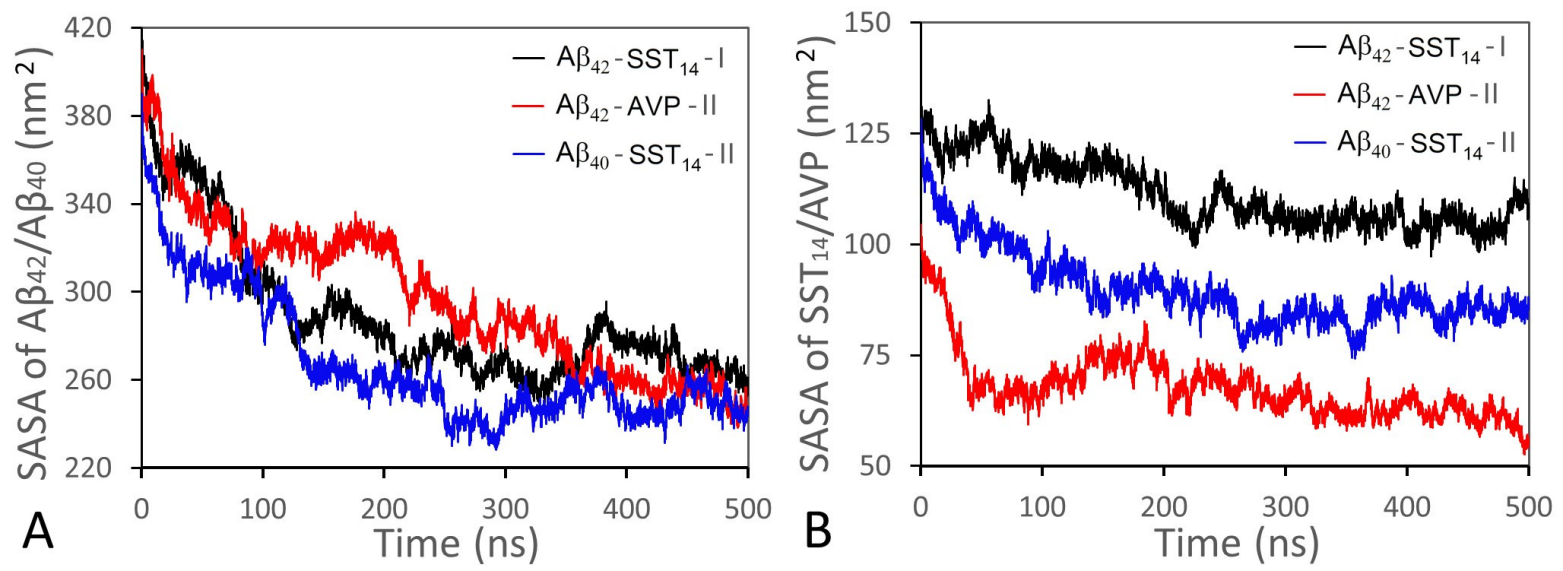
Total SASA of Aβ chains (A) and small cyclic peptides (B) for systems Aβ_42_-SST_14_-I (black lines), Aβ_42_-AVP-II (red lines), and Aβ_40_-SST_14_-II (blue lines) during 500 ns production MD simulations.

The solvent accessible surface area (SASA) is an important quantitative indicator of the oligomerization process. Fig 8 shows the evolution of total SASAs for Aβ chains and small cyclic peptides for three representative systems Aβ_42_-SST_14_-I, Aβ_42_-AVP-II, and Aβ_40_-SST_14_-II during 500 ns production MD simulations. Consistent with the ongoing aggregation in these systems, total SASAs of Aβ chains decrease significantly in the course of the simulations (Fig 8A). Although the greatest decrease occurs over the first 200–250 ns, a tendency of continuing collapse persists over the entire 500 ns interval. Total SASAs of small cyclic peptides also exhibit a steady decrease (Fig 8B). At the beginning of the simulations SASAs of SST_14_ are close across the Aβ_42_-SST_14_-I and Aβ_40_-SST_14_-II systems; however subsequently SST_14_ tends to exhibit a greater SASA in the Aβ_42_-SST_14_-I system than in the Aβ_40_-SST_14_-II system. There are two reasons for that. First, one or more SST_14_ molecules always remain free in the Aβ_42_-SST_14_-I system. Second, from our analysis of the trajectories it follows that the SST_14_ molecules tend to occupy peripheral regions of aggregates in Aβ_42_-SST_14_ system, whereas in Aβ_40_-SST_14_ system, they are often found in the interior of aggregates. The total SASA of AVP is less than that of SST_14_ in either system due to smaller size of the AVP molecules.

Table 4 lists average hydrophobic and hydrophilic SASAs for all the peptides in each of the three systems Aβ_42_-SST_14_, Aβ_42_-AVP and Aβ_40_-SST_14_ during the first and the last 5 ns of 500 ns long MD simulations, and S5 Table provides similar data for SST_14_ and AVP molecules separately. Additional details of the SASAs in individual trajectories are given in S6 Table. As one could expect, the hydrophobic SASA is less than the hydrophilic counterpart in all systems at all stages of the production MD simulations, since the aggregation process facilitates burying of hydrophobic groups. As Table 4 indicates, exposures of both hydrophobic and hydrophilic groups to the solvent have decreased considerably in the course of the simulations. The relative decrease amounts to approximately 28–37% of the initial SASA’s values in each of the three systems. Upon averaging over three trajectories for each of the systems Aβ_42_-SST_14_, Aβ_42_-AVP and Aβ_40_-SST_14_, the total hydrophilic SASAs are quite uniform across all three systems during the first 5 ns of MD simulations, with a slightly increased contribution of hydrophilic residues in Aβ_42_-AVP (Table 4). In contrast, the initial hydrophobic SASAs of the three systems exhibit diverging trends across the three systems. At the beginning of MD simulations, total hydrophobic SASAs in all Aβ_42_-SST_14_ trajectories are higher than in any of Aβ_40_-SST_14_ trajectories (S6 Table), which can be explained by the presence of the two hydrophobic C-terminal residues in Aβ_42_. On the other hand, the initial hydrophobic SASAs in both SST_14_ containing systems are pronouncedly higher than in all Aβ_42_ -AVP trajectories (Tables 4 and S6), which we attribute to the larger hydrophobic SASA of SST_14_ molecules compared to AVP molecules (S5 Table).

**Table 4:** Hydrophobic and hydrophilic SASA in each system averaged over three trajectories during the first and the last 5 ns of MD simulations.

Systems	First 5 ns (nm^2^)		Last 5 ns (nm^2^)		Difference (%)	
	Hydrophobic	Hydrophilic	Hydrophobic	Hydrophilic	Hydrophobic	Hydrophilic
**Aβ**_**42**_**-SST**_**14**_	220.4 (±8.3)	290.7 (±6.8)	147.9 (±5.8)	210.7 (±16.9)	33	28
**Aβ**_**42**_**-AVP**	187.0 (±8.2)	296.6 (±6.3)	118.7 (±9.2)	186.3 (±17.6)	37	37
**Aβ**_**40**_**-SST**_**14**_	205.6 (±5.5)	288.1 (±4.2)	130.5 (±2.0)	205.1 (±13.9)	37	29

In the course of the MD simulations, the Aβ_42_-SST_14_ system exhibited the smallest relative decrease in hydrophobic SASA (33%), consistent with a smaller size of aggregates and greater solvent exposure of Aβ chains and SST_14_ molecules in these systems (Table 4). The reduction in hydrophobic SASA in both Aβ_42_-AVP and Aβ_40_-SST_14_ systems is approximately 37% despite a relatively weak hydrophobicity of AVP. Together with a pronounced reduction in exposure of hydrophilic residues in Aβ_42_-AVP, this may suggest more efficient collapse in Aβ_42_-AVP system in comparison to Aβ_40_-SST_14_ system.

Average hydrophobic and hydrophilic SASAs calculated for SST_14_ alone (S5 Table) exhibit consistent trends with those in Table 4. Initially, SST_14_’s hydrophobic and hydrophilic SASAs are close in both systems. In the course of the subsequent simulations, the decrease in both hydrophilic and hydrophobic SASA of SST_14_ is less pronounced in Aβ_42_-SST_14_ system than in Aβ_40_-SST_14_ system. In the case of AVP, the relative decrease in hydrophobic and hydrophilic SASA of 49% and 43%, respectively, is the greatest of all systems. In addition, the high initial hydrophilicity in AVP results in a three times greater absolute reduction of hydrophilic SASA in comparison to the hydrophobic one.

Summarising, the greater initial hydrophobicity of the Aβ_42_-SST_14_ system might result in substantial hydrophobic collapse; however, the observed decrease in solvent exposure of hydrophobic residues in the Aβ_42_-SST_14_ system was relatively weak. Contrary to the expectations, a higher decrease of hydrophobic SASA during the MD simulations was observed in Aβ_40_-SST_14_ system instead. The Aβ_42_-AVP system exhibited the strongest reduction in solvent exposure of hydrophilic residues, suggesting a more important role of electrostatic interactions in the course of aggregation in these systems.

## Discussion

We have conducted all-atom MD simulations of early aggregation events at the onset of oligomerization in mixtures of Aβ peptides and small cyclic peptides, Aβ_42_–SST_14_, Aβ_42_–AVP, and Aβ_40_–SST_14_ in water. Initially, the Aβ peptides and small cyclic peptides were sparsely positioned with respect to each other. Aggregation of the Aβ peptides, accompanied by a pronounced decrease in solvent exposure of both hydrophobic and hydrophilic groups, went on throughout the 500 ns long MD runs. In the course of the simulations, all systems developed self-assembled aggregates containing Aβ chains and small cyclic peptides. Consistent with published experiments [10,11] and MD simulations [15,16] of Aβ aggregation in the absence of small cyclic peptides, the self-assembled aggregates observed in this study exhibit largely unstructured morphologies without pronounced alignment of the chains. Although such unstructured morphologies indicate a considerable influence of hydrophobic collapse onto the oligomerization, the observed build-up of hydrogen bonding accompanied by development of stable β-sheet content also suggests a potential tendency toward β-conversion.

Overall, self-assembled Aβ_42_–SST_14_ aggregates are compact and well-correlated dynamically. However, they tend to be smaller in size than aggregates formed in the other systems; their hydrophobic groups exhibit the highest solvent accessibilities in average by the end of the 500 ns simulation; and they develop less hydrogen bonds across Aβ_42_ peptides and SST_14_ molecules in comparison to the other systems. The replacement of SST_14_ with AVP molecules produced larger aggregates. In addition, the Aβ_42_–AVP system tended to develop more hydrogen bonds than the other systems, which is especially true for cross-species hydrogen bonding. In the course of the 500 ns simulations, the AVP molecules developed almost 3 times more HBs with Aβ_42_ than the SST_14_ molecules did. Both hydrophobic and hydrophilic SASA are the lowest in the Aβ_42_–AVP systems in average after 500 ns simulations. The two Aβ_42_-containing systems exhibited similar tendencies of Aβ’s C-termini to remain at the aggregate’s surface, although C-terminal flexibility tended to be greater in AVP-containing systems than in SST_14_-containing systems. The most pronounced differences between the Aβ_42_–SST_14_ and Aβ_40_–SST_14_ systems concern the morphologies of the self-assembled aggregates. Unlike the Aβ_42_–SST_14_ system, the Aβ_40_–SST_14_ system formed large aggregates that contained a domain of correlated motion surrounded by relatively weakly correlated parts. In the Aβ_40_–SST_14_ system the C-termini are buried more often than in the other two systems, and they exhibit the lowest average C-terminal flexibility. According to a recent report [21], such morphological differences may lead to different pathways at later stages of oligomerization.

Our examination of inter-molecular contacts that exhibited the strongest dynamics correlations in average over the last 20 ns of the simulations revealed differences in the interactions of SST_14_ and AVP molecules with Aβ peptides. In Aβ_42_-SST_14_ aggregates, highly correlated regions tended to involve residues K4, N5, F7, W8, and T10 of SST_14_, in agreement with biochemical observations [28]. These residues were found to interact primarily with the residues 28–35 of Aβ_42_, although contacts with its C-terminus were also detected (K4-V40 and W8-V40). The interactions tended to involve hydrophobic contacts, although backbone-backbone hydrogen bonds were also present. In the Aβ_42_-AVP aggregates, strong correlations involved predominantly contacts between N-terminal AVP resides C1, Y2, and Q4, and residues 20–23 of Aβ_42_. In the Aβ_40_-SST_14_ aggregates, T10 and F11 of SST_14_ were found to interact with C-terminal residues 35–40 of Aβ_40_. Hydrogen bonds are found frequently between strongly correlated residues in systems Aβ_42_-AVP and Aβ_40_-SST_14_.

At a coarser level, we observe a tendency of SST_14_ molecules to develop hydrophobic contacts with surfaces of Aβ_42_ aggregates such that both N- and C- terminal arms of SST_14_ face the solvent. This may prevent hydrophobicity-driven coalescence the Aβ_42_ aggregates, explaining the relatively small size of aggregates in the Aβ_42_-SST_14_ system. Distinct from SST_14_, AVP molecules tend to penetrate inside the Aβ_42_ aggregates, where they develop extensive hydrogen bonding with central regions of Aβ_42_ peptides. When SST_14_ interacts with small Aβ_40_ aggregates, the morphologies resemble those of Aβ_42_-SST_14_, whereas large Aβ_40_ aggregates tend to incorporate SST_14_ molecules resembling the interaction of AVP with Aβ_42_ aggregates.

Based on the results obtained in this study, the experimentally observed differences in the aggregation behaviours of Aβ_42_–SST_14_, Aβ_42_–AVP, and Aβ_40_–SST_14_ mixtures [28,29] may be attributed to the different interaction of the small cyclic peptides with each of the Aβ peptides and the solvent.

First, the tendency of SST_14_ to develop predominantly hydrophobic contacts with residues 28–35 and the C-terminus of Aβ_42_ favors morphologies where several Aβ_42_ chains form small aggregates compacted by Aβ-Aβ hydrogen bonding, and carrying SST_14_ molecules attached to their surfaces. This surface attachment prevents the coalescence of small Aβ_42_ aggregates into bigger structures. Within the time regimes of the present MD study, this results in a prevalence of multiple small Aβ_42_ aggregates. The resulting delay in hydrophobicity-driven coalescence may explain the extended lag phase of Aβ_42_ aggregation observed experimentally [28]. In contrast, AVP molecules preferentially bind to central regions of Aβ_42_ peptides, and are prone to develop extensive inter-species hydrogen bonding. As a result, large Aβ_42_ aggregates self-assemble quickly and contain inserted AVP molecules that strengthen connections within the aggregates. The binding of SST_14_ to Aβ_40_ tended to rely on strong connections with the C-terminal region of the Aβ_40_ chains. Combined with the propensity of the Aβ_40_-SST_14_ system to develop inter-species hydrogen bonding, this facilitated insertion of SST_14_ into the interior of Aβ_40_ aggregates.

Second, differences in hydrophobicity of the three systems result in different surface reactivities of the aggregates. Clearly, the presence of SST_14_ imparts the aggregates with a greater hydrophobicity. The resulting “sticky surface” effect is enhanced even more due to the location of both SST_14_ molecules and hydrophobic C-termini, preferentially at the surface of the aggregates. In the Aβ_42_–AVP system hydrophobic C-termini are also exposed and flexible. However, the “sticky surface” effect is lessened by a lower hydrophobicity of AVP and a lesser surface area due to the larger size of the aggregates. In the Aβ_40_-SST_14_ system, the insertion of SST_14_ molecules into the interior of the aggregates combined with the reduced number of exposed hydrophobic C-terminal residues plays a role.

Summarising, our observations suggest that mixtures of Aβ_42_ and SST_14_, although pronouncedly more hydrophobic than their Aβ_42_-AVP and Aβ_40_-SST_14_ counterparts, tend to aggregate slower than the other two mixtures retaining extensive “sticky surface” areas exposed for longer times. This may explain the increased pathogenicity of Aβ_42_, but not Aβ_40_ peptides, in the presence of SST_14_, but not AVP molecules.

## Methods

### Modeling structures

Each system investigated in this study contained eight randomly positioned monomeric Aβ_42_ or Aβ_40_ peptides and eight randomly positioned small cyclic peptides, somatostatin-14 (SST_14_) or arginine vasopressin (AVP), as listed in Table 5. For Aβ_42_ peptides, the sequence _1_DAEFRHDSGYEVHHQKLVFFAEDVGSNKGAIIGLMVGGVVIA_42_ was used, and for Aβ_40_ peptides the two C-terminal residues were excluded. The sequences _1_AGCKNFFWKTFTSC_14_ and _1_CYFENCPRG_9_-NH_2_ were used for SST_14_ and AVP, respectively, to match the conditions of the experiments [28,29]. As starting coordinates for these monomeric units, we used the coordinates of chains A–F of the NMR-derived Aβ_1–42_ hexamer from PDB ID 2NAO [36] and the coordinates of chains A-H of the Aβ_1–40_ nanomer from PDB ID 2M4J [37], which were equilibrated before modeling. For small cyclic peptides, we used the coordinates of chain A of somatostatin-14 with S-S bond from PDB ID 2MI1 [38], and coordinates of chain B of vasopressin from PDB ID 1YF4 [39]. Using the Accelrys VS [40] and VMD [41] packages, we built the starting systems to contain eight monomeric Aβ_1–40/42_ units from 2M4J/2NAO.pdb, and eight SST14/AVP molecules by randomly rotating and shifting the units against each other. All peptides were randomly placed in the simulation box such that distances between their surfaces were longer than 4 Å. This resulted in sparsely positioned units with a concentration of approximately 4 mM for Aβ, SST_14_, and AVP each.

**Table 5:** List of the systems studied.

System	Details of preparation	Number of MD trajectories
**Aβ**_**42**_**-SST**_**14**_**-I/III**	Eight Aβ_1–42_ chains 2NAO [36], pre-equilibrated and mixed with eight SST_14_ chains 2MI1 [38]; then equilibrated again.	3
**Aβ**_**42**_**-AVP-I/III control**	Eight Aβ_1–42_ chains 2NAO [36], pre-equilibrated and mixed with eight AVP chains 1YF4 [39]; then equilibrated again.	3
**Aβ**_**40**_**-SST**_**14**_**-I/III**	Eight Aβ_1–40_ chains 2M4J [37], pre-equilibrated and mixed with eight SST_14_ chains 2MI1 [38]; then equilibrated again.	3

Minimizations, equilibrations, and production MD simulations were carried out using the GROMACS v5.0.7 package [42]. The AMBER99SB-ILDN forcefield [43] was used for protein atoms. Then, we minimized each set of 16 randomly positioned units using a steepest descent algorithm for energy in vacuum, and added the solvent using the optimized general-purpose 4-point OPC water model [44], as well as counterions Na+/Cl− to electro-neutralize the systems. Subsequent solvent minimizations involved decreasing position restraints on non-hydrogen protein atoms, as well as heating with the Berendsen thermostats. NVT-equilibrations were performed on all the systems. To collect sufficient statistics on the systems’ dynamics, we ran independent MD trajectories for each of the systems, as listed in Table 5. The same sets of starting coordinates of atoms were used in each of the trajectories, whereas the seed numbers were varied when generating the initial Maxwell distributions of atom velocities. The three MD trajectories were labeled as “I”, “II” and “III”.

### Molecular dynamics simulations

The production MD simulations, as well as the last equilibration step, were conducted at a temperature of 310 K and a pressure of 1 atm using isotropic pressure coupling (NPT ensemble), the Verlet cut-off scheme for neighbour searching, the Particle-Mesh Ewald treatment of electrostatics, a twin-range cut-off for van der Waals interactions, and bond lengths restrained through the linear constant solver (LINCS) algorithm with a fourth order of expansion. A V-rescale scheme was used for temperature coupling, and the Parrinello-Rahman algorithm was employed for pressure coupling applying the scaling to the center of mass of reference coordinates. Production MD simulations were performed for 100 ns for 9 systems containing 16 chains each (see Table 5). 2 fs time steps were used, and snapshots were saved every 20 fs in order to analyze the essential collective dynamics of the systems.

Structural analysis of the trajectories, including assessment of the secondary structure, the number of hydrogen bonds, and solvent accessible areas, has been done using the GROMACS scripts [42] and the VMD package [41]. For graphical representation, the VMD and Accelrys VS packages were utilized.

### Essential collective dynamics analysis

To analyze the dynamics of the aggregates in greater depth we employed the novel essential collective dynamics (ECD) method [16,31–35], which stemmed from a recently developed statistical-mechanical framework [31,32]. In this framework, a macromolecule or supramolecular complex is described by generalized Langevin equations with a set of essential collective coordinates identified as principal eigenvectors of a covariance matrix, which in turn is calculated by applying the principal component analysis (PCA) on MD trajectories. It has been demonstrated [31,32] that persistent correlations between atoms’ motion (dynamics) can be calculated from a projected all-atom image of the system in a multi-dimensional space of the essential collective coordinates, as described in details elsewhere [16,31–33]. A suite of dynamics descriptors has been derived within this framework, including pair correlation maps [16,33,35], dynamics domains of correlated motion [16,31,33,34], and main-chain flexibilities [16,33–35]. The method has been validated extensively against NMR [31–34,45] and X-ray [33,46] structural data, and the corresponding descriptors were demonstrated to predict accurately the persistent dynamics trends from short fragments of MD trajectories without exhaustive conformational sampling [31,32]. In this study, we applied the ECD method with a set of 10 principal eigenvectors, which were sufficient to sample more than 95% of the total displacement in the systems considered. The ECD descriptors were calculated from multiple 0.2 ns long segments of the MD trajectories, and averaged over pertinent segments of production MD simulations, as specified in Results and Discussion. The pair correlation descriptors displayed in Fig 4 have been derived from the projected images of Cα atoms in the respective aggregates as specified earlier [16,33,34,45]. The dynamics domains of correlation motion, which represent relatively rigid parts of the aggregate composed of atoms moving coherently (Fig 5) were identified by applying the clustering technique described in earlier reports [31,33,34,45] with a threshold of 0.008 to the projected all-atom images of all systems. The main-chain flexibility profiles (Fig 6) illustrate the level of dynamic coupling of main-chain atoms with the entire aggregate, which in turn is represented by the centroid of the projected all-atom images of the aggregate.

## Supporting information

S1 Fig Close-ups after equilibration at the beginning of production MD simulations illustrated by examples from representative trajectories Aβ_42_-SST_14_-I (A), Aβ_42_-AVP-II (B), and Aβ_40_-SST_14_ II (C). All main-chains are shown as ribbons. The C-terminal residues 36-40/42 are colored blue. SST_14_ molecules (A,C) and AVP molecules (B) are depicted in atomic details and colored red. Hydrophobicity of all chains is color-mapped onto solvent accessible surfaces, where blue indicates hydrophobic residues and red indicates hydrophilic residues.
(TIF)

S2 Fig Self-assembled aggregates in trajectories Aβ_42_-SST_14_- I (A); Aβ_42_-AVP-II (B); and Aβ_40_-SST_14_-II (C) after 500 ns simulations. In Aβ_42_ and Aβ_40_ chains, random coils are colored white, β-strands are colored yellow, turns are colored cyan, and α helices are colored purple; the C-terminal residues 36-40/42 of Aβ peptides are colored blue. The SST_14_ molecules (A,C) and AVP molecules (B) are depicted in atomic detail and colored red.
(TIF)

S3 Fig The number of hydrogen bonds across Aβ chains and small cyclic peptides during 500 ns production simulations. (A)–the numbers of hetero-molecular bonds as functions of time in all nine trajectories. Aβ_42_-SST_14_ systems are shown with shades of red, Aβ_42_-AVP–with shades of blue, and Aβ_40_-SST14 –with shades of green. (B)–the dependencies for Aβ_42_-SST_14_-I (red line), Aβ_42_-AVP-II (blue line), and Aβ_40_-SST_14_-II (green line) with solid dots indicating local maxima and minima where the snapshots presented in Fig 3 were taken.
(TIF)

S4 Fig Secondary structure evolution for trajectories Aβ_42_-SST_14_-I (A), Aβ_42_-AVP-II (B) and Aβ_40_-SST_14_-II (C) during the 500 ns long production MD simulations. β-sheets/bridges are shown with yellow/dark yellow color, α/3-π-helices–with purple/blue, turns–with green, and random coils–with white. The Y-axis represents residues in chains A-P.
(TIF)

S5 Fig Percentages of β-sheet (top row), β-bridge (middle row), and α/3-π-helix (bottom row) content in each of the three MD trajectories for systems Aβ_42_-SST_14_, Aβ_42_-AVP, and Aβ_40_-SST_14_ as functions of time.
(TIF)

S6 Fig Aggregates observed in systems Aβ42-SST14-I (A), Aβ42-AVP-II (B) and Aβ40-SST14-II (C) after 500 ns of simulations. In (A), chains A, B and E are colored yellow; chains C, D and F are colored cyan; and chains G and H are colored purple. In (B) and (C), chains A to H are colored yellow, cyan, purple, lime, mauve, ochre, iceblue and black, respectively. All SST_14_ and AVP molecules are colored red.
(TIF)

S1 Table Number of hydrogen bonds between and across Aβ chains and small cyclic peptides (SCP), and total number of hydrogen bonds in each system in average over the first and the last 20 nanoseconds of 500 ns long MD runs. (XLSX)

S2 Table Population of β-bridges in all trajectories averaged over the last 20 ns of the 500 ns long MD simulations, their averages over three trajectories for each system, the averages over the entire 500 ns simulations, and standard deviations of the data. The data are given in % of occupied Aβ residues.
(XLSX)

S3 Table Population of α-helices and 3-π-helices in all trajectories averaged over the last 20 ns of the 500 ns long MD simulations, their averages over three trajectories for each system, the averages over the entire 500 ns simulations, and standard deviations of the data. The data are given in % of occupied Aβ residues.
(XLSX)

S4 Table ECD main-chain flexibility of C-terminus in each Aβ_42_ or Aβ_40_ chain in average over the last 20 ns of each MD trajectory. (XLSX)

S5 Table Hydrophobic and hydrophilic SASA of the small cyclic peptides in each system averaged over three trajectories, during the first and the last 5 ns of MD simulations, and the corresponding differences. (XLSX)

S6 Table Hydrophobic and hydrophilic SASA in average over the first and the last 5 ns of MD simulations, and the corresponding differences, for all the systems. (XLSX)
