# Supplementary figures and images for: Aggregation of Aβ40/42 chains in the presence of cyclic neuropeptides investigated by molecular dynamics simulations
Source: PLoS Comput Biol. 2021 Mar 12;17(3):e1008771. doi: 10.1371/journal.pcbi.1008771 (PMC7990313; doi:10.1371/journal.pcbi.1008771)

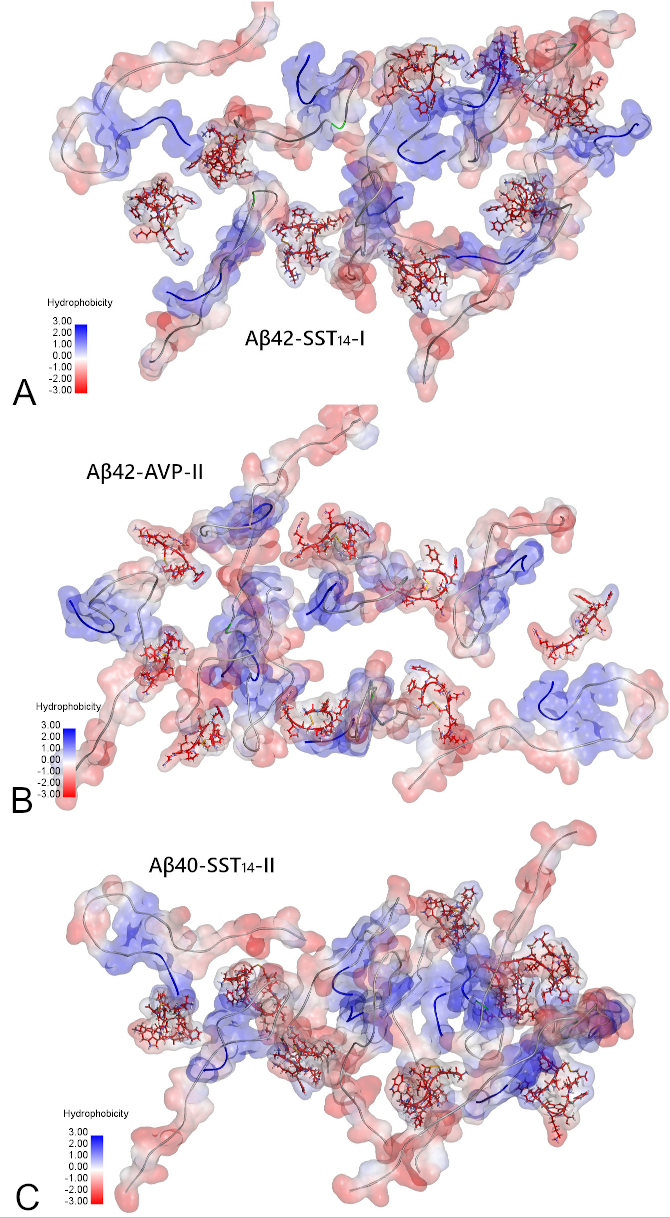

Supplement: S1 Fig — Close-ups after equilibration at the beginning of production MD simulations illustrated by examples from representative trajectories Aβ42-SST14-I (A), Aβ42-AVP-II (B), and Aβ40-SST14 II (C). All main-chains are shown as ribbons. The C-terminal residues 36-40/42 are colored blue. SST14 molecules (A,C) and AVP molecules (B) are depicted in atomic details and colored red. Hydrophobicity of all chains is color-mapped onto solvent accessible surfaces, where blue indicates hydrophobic residues and red indicates hydrophilic residues. (TIF) [file pcbi.1008771.s001.tif]

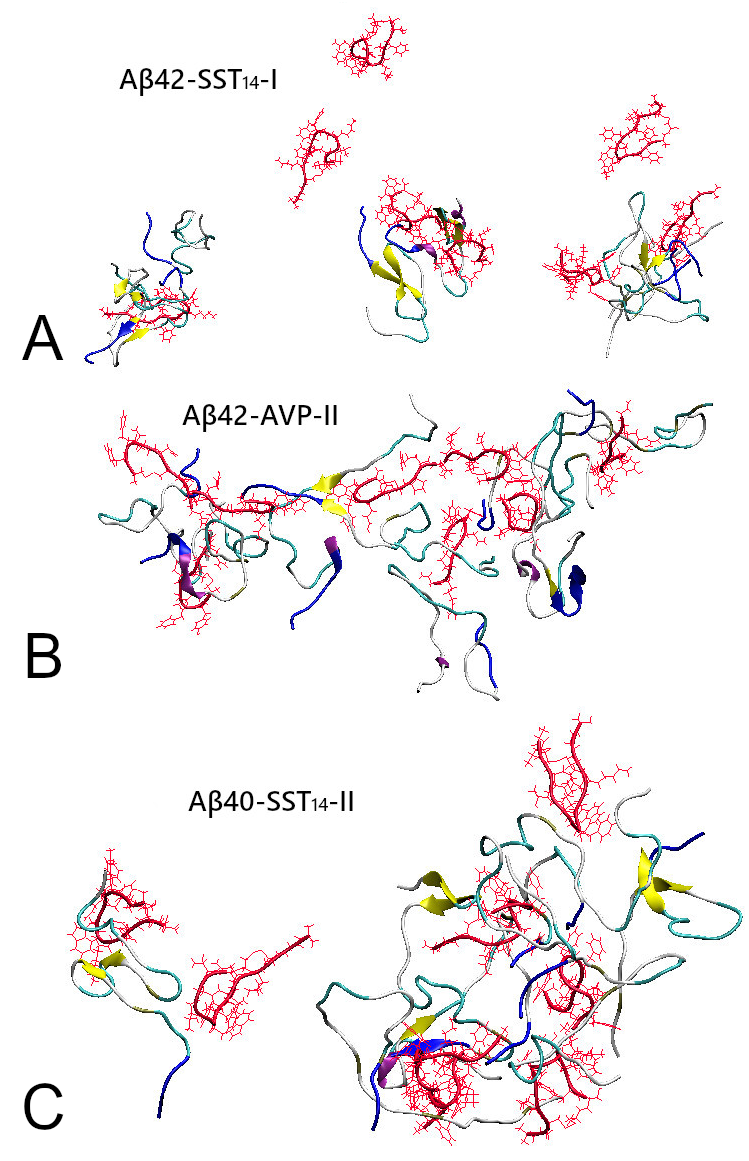

Supplement: S2 Fig — Self-assembled aggregates in trajectories Aβ42-SST14- I (A); Aβ42-AVP-II (B); and Aβ40-SST14-II (C) after 500 ns simulations. In Aβ42 and Aβ40 chains, random coils are colored white, β-strands are colored yellow, turns are colored cyan, and α helices are colored purple; the C-terminal residues 36-40/42 of Aβ peptides are colored blue. The SST14 molecules (A,C) and AVP molecules (B) are depicted in atomic detail and colored red. (TIF) [file pcbi.1008771.s002.tif]

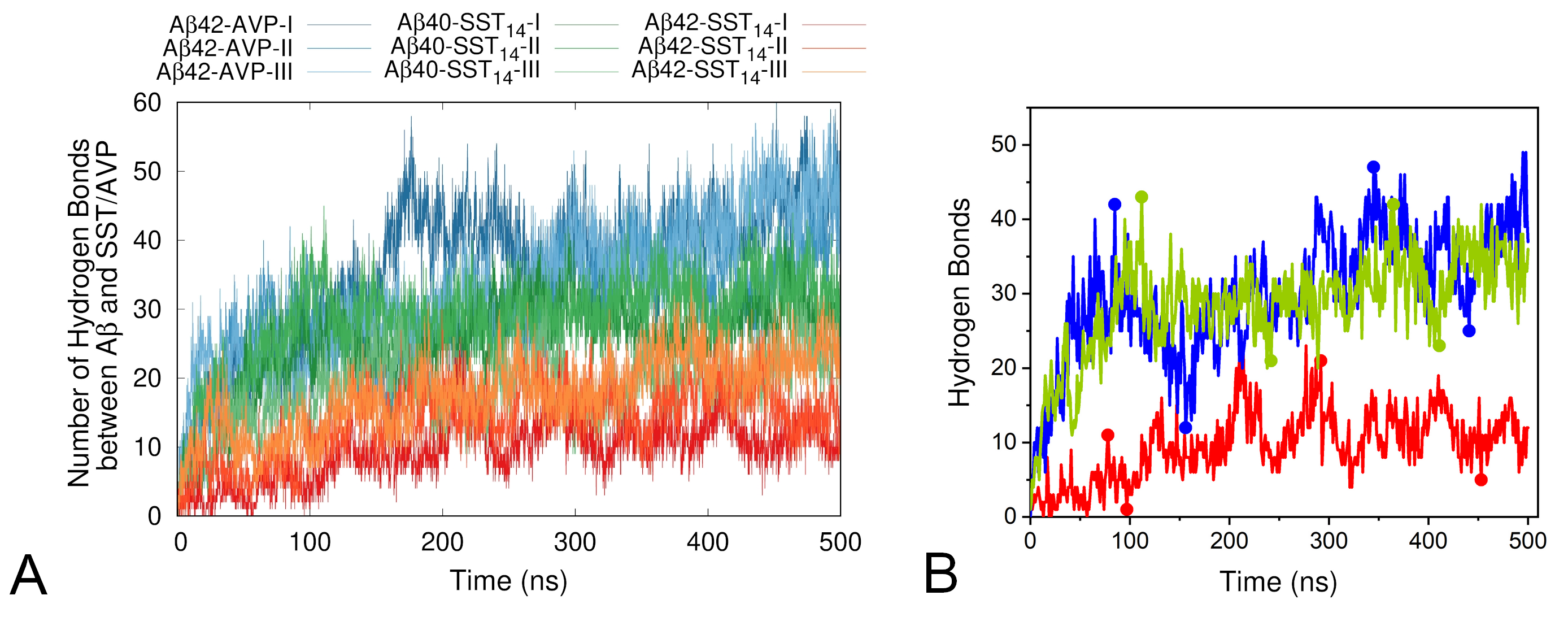

Supplement: S3 Fig — The number of hydrogen bonds across Aβ chains and small cyclic peptides during 500 ns production simulations. (A)–the numbers of hetero-molecular bonds as functions of time in all nine trajectories. Aβ42-SST14 systems are shown with shades of red, Aβ42-AVP–with shades of blue, and Aβ40-SST14 –with shades of green. (B)–the dependencies for Aβ42-SST14-I (red line), Aβ42-AVP-II (blue line), and Aβ40-SST14-II (green line) with solid dots indicating local maxima and minima where the snapshots presented in Fig 3 were taken. (TIF) [file pcbi.1008771.s003.tif]

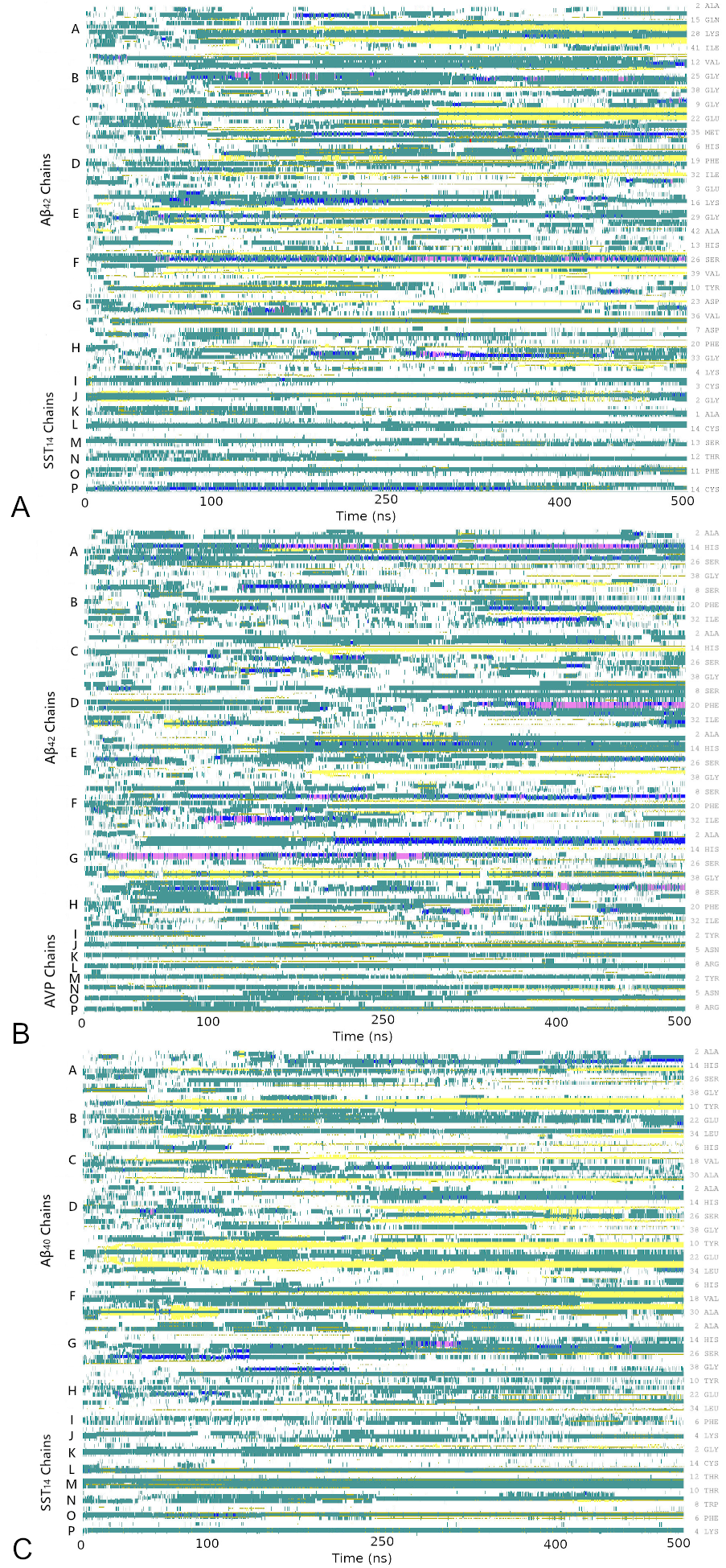

Supplement: S4 Fig — Secondary structure evolution for trajectories Aβ42-SST14-I (A), Aβ42-AVP-II (B) and Aβ40-SST14-II (C) during the 500 ns long production MD simulations. β-sheets/bridges are shown with yellow/dark yellow color, α/3-π-helices–with purple/blue, turns–with green, and random coils–with white. The Y-axis represents residues in chains A-P. (TIF) [file pcbi.1008771.s004.tif]

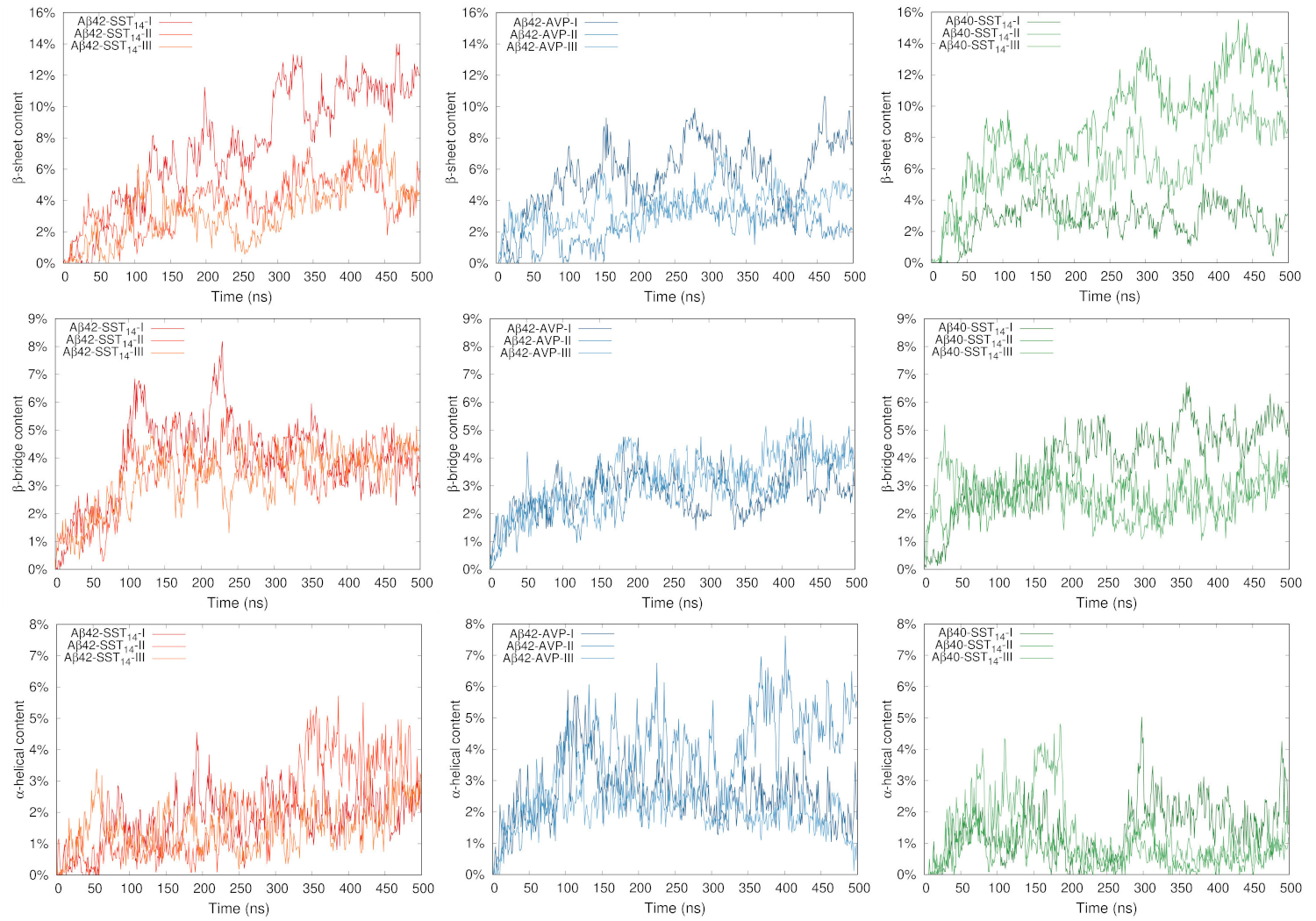

Supplement: S5 Fig — Percentages of β-sheet (top row), β-bridge (middle row), and α/3-π-helix (bottom row) content in each of the three MD trajectories for systems Aβ42-SST14, Aβ42-AVP, and Aβ40-SST14 as functions of time. (TIF) [file pcbi.1008771.s005.tif]

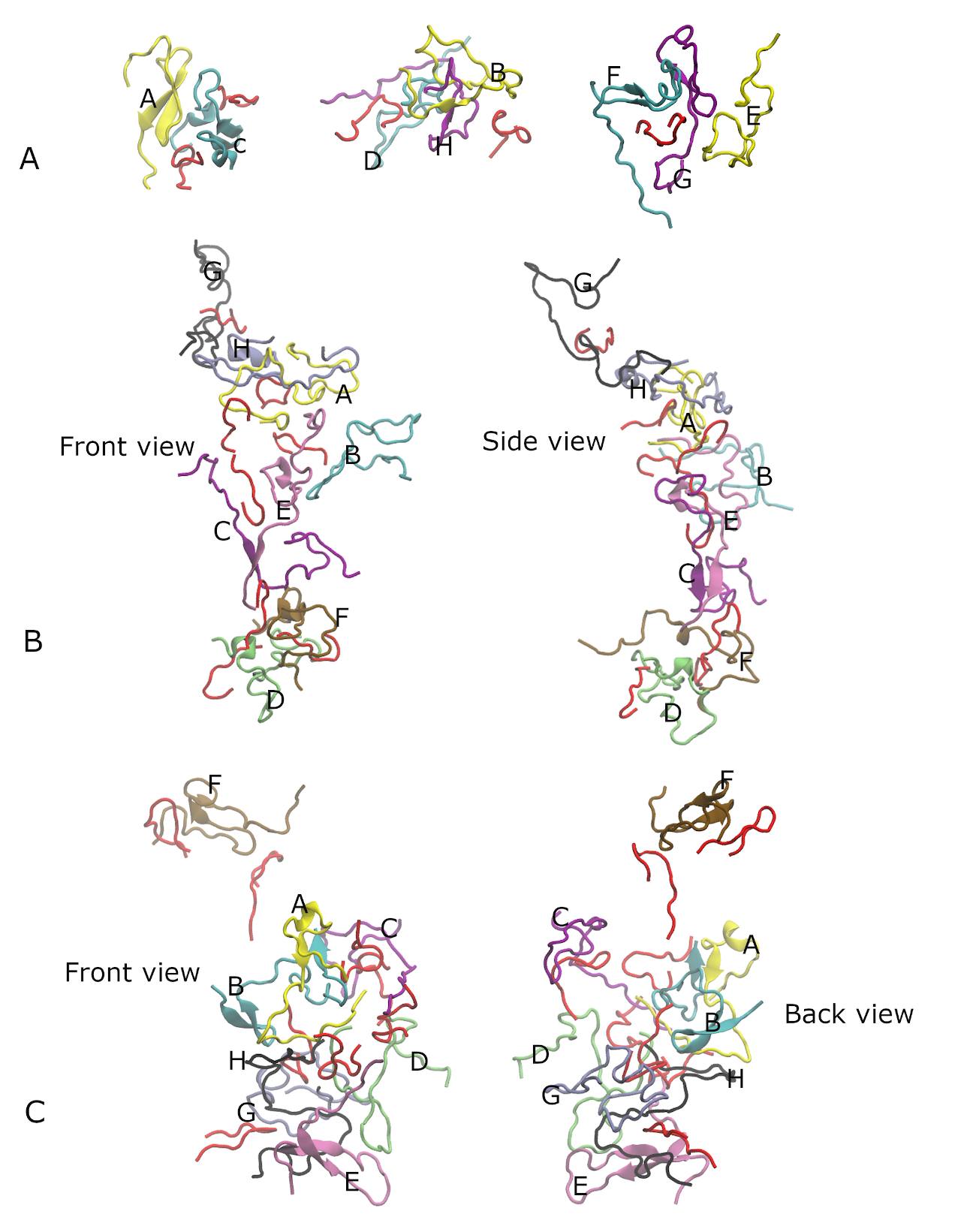

Supplement: S6 Fig — Aggregates observed in systems Aβ42-SST14-I (A), Aβ42-AVP-II (B) and Aβ40-SST14-II (C) after 500 ns of simulations. In (A), chains A, B and E are colored yellow; chains C, D and F are colored cyan; and chains G and H are colored purple. In (B) and (C), chains A to H are colored yellow, cyan, purple, lime, mauve, ochre, iceblue and black, respectively. All SST14 and AVP molecules are colored red. (TIF) [file pcbi.1008771.s006.tif]
